# Effects of Anticancer Agent P-bi-TAT on Gene Expression Link the Integrin Thyroid Hormone Receptor to Expression of Stemness and Energy Metabolism Genes in Cancer Cells

**DOI:** 10.3390/metabo12040325

**Published:** 2022-04-04

**Authors:** Gennadi V. Glinsky, Kavitha Godugu, Thangirala Sudha, Mehdi Rajabi, Sridar V. Chittur, Aleck A. Hercbergs, Shaker A. Mousa, Paul J. Davis

**Affiliations:** 1 Institute of Engineering in Medicine, University of California San Diego, San Diego, CA 92037, USA; 2 Pharmaceutical Research Institute, Albany College of Pharmacy and Health Sciences, One Discovery Drive, Rensselaer, NY 12144, USA; kavitha.godugu@acphs.edu (K.G.); sudha.thangirala@acphs.edu (T.S.); m.rajabi.s@gmail.com (M.R.); shaker.mousa@acphs.edu (S.A.M.); 3 Center for Functional Genomics, University at Albany, Rensselaer, NY 12144, USA; schittur@albany.edu; 4 Cleveland Clinic, Cleveland, OH 44195, USA; hercbergs@gmail.com; 5 Department of Medicine, Albany Medical College, Albany, NY 12208, USA

**Keywords:** ATP synthase, cancer cells, mitochondria, glioblastoma, integrin αvβ3, NADH dehydrogenase, tetrac, thyroid hormones

## Abstract

Chemically modified forms of tetraiodothyroacetic acid (tetrac), an L-thyroxine derivative, have been shown to exert their anticancer activity at plasma membrane integrin αvβ3 of tumor cells. Via a specific hormone receptor on the integrin, tetrac-based therapeutic agents modulate expression of genes relevant to cancer cell proliferation, survival and energy metabolism. P-bi-TAT, a novel bivalent tetrac-containing synthetic compound has anticancer activity in vitro and in vivo against glioblastoma multiforme (GBM) and other types of human cancers. In the current study, microarray analysis was carried out on a primary culture of human GBM cells exposed to P-bi-TAT (10^−6^ tetrac equivalent) for 24 h. P-bi-TAT significantly affected expression of a large panel of genes implicated in cancer cell stemness, growth, survival and angiogenesis. Recent interest elsewhere in ATP synthase as a target in GBM cells caused us to focus attention on expression of genes involved in energy metabolism. Significantly downregulated transcripts included multiple energy-metabolism-related genes: electron transport chain genes ATP5A1 (ATP synthase 1), ATP51, ATP5G2, COX6B1 (cytochrome c oxidase subunit 6B1), NDUFA8 (NADH dehydrogenase (ubiquinone) FA8), NDUFV2I and other NDUF genes. The NDUF and ATP genes are also relevant to control of oxidative phosphorylation and transcription. Qualitatively similar actions of P-bi-TAT on expression of subsets of energy-metabolism-linked genes were also detected in established human GBM and pancreatic cancer cell lines. In conclusion, acting at αvβ3 integrin, P-bi-TAT caused downregulation in human cancer cells of expression of a large number of genes involved in electron transport and oxidative phosphorylation. These observations suggest that cell surface thyroid hormone receptors on αvβ3 regulate expression of genes relevant to tumor cell stemness and energy metabolism.

## 1. Introduction

Acting at the thyroid hormone analogue receptor on the distal aspect or head of the extracellular domain of cell membrane integrin αvβ3, L-thyroxine (T4) at physiological concentrations stimulates cancer cell and endothelial cell proliferation [1,2,3,4]. Expression of αvβ3 is generous in malignant cells and blood vessel cells but not in non-dividing normal cells; thus, T4 is minimally active in such cells. In normal, nonmalignant cells, 3,5,3′-triiodo-L-thyronine (T3) is the principal form of thyroid hormone, acting at mitochondria to regulate cellular respiration and in the nucleus via specific thyroid hormone receptors (TRs) to regulate gene expression controlling cell division and a panel of other cell functions [1].

Tetraiodothyroacetic acid (tetrac) is a naturally occurring derivative of T4 that displaces T4 from the thyroid hormone analogue receptor site on integrin αvβ3 and also initiates a number of intracellular actions via the integrin in the absence of T4 [3]. Chemically modified forms of tetrac, Nanotetrac and P-bi-TAT, are synthesized as relatively large, nano-scale size molecules that enhance tetrac activity at the integrin in terms of reduced cancer cell proliferation and induced apoptosis [2,3,4]. Known mechanisms of these anticancer actions primarily reflect changes in expression of specific genes [2,3]. P-bi-TAT [5,6,7], a first-in-class divalent tetrac-based nanopharmaceutical, has been shown to markedly inhibit growth of human GBM cells in mouse xenograft models [7] causing downregulation of expression of families of genes that are critical to cancer cell growth and survival [8]. The molecular size and structure of P-bi-TAT limit its uptake by cancer cells [7], restrict its access to cells’ interior and abrogate actions in nuclei when the residual amount of agent is internalized by cancer cells.

Shi et al. [9] recently emphasized the mitochondrion as a focus of anticancer activity in glioblastoma (GBM) cells, allografts and xenografts. They demonstrated the effectiveness of gboxin, a novel small molecule benzimiazolinium, in targeting the activity of ATP synthase in GBM mitochondria. Gboxin did not affect several normal cell models. We have shown that tetrac-containing agents have activity against a variety of cancer cells [10,11,12,13,14,15] and, specifically, that P-bi-TAT is effective against xenografts of human GBM cells [7]. The actions of P-bi-TAT are limited to cancer cells and rapidly dividing endothelial cells [3,4]. These preclinical findings have potential significant translational implications because it remains clear that more effective clinical anti-GBM therapy is urgently needed [16,17,18,19].

In the current study, we report expanded observations on mechanisms of anticancer activities of P-bi-TAT in a primary culture of human GBM cells (PC-GBM) in terms of modulation by the drug of gene expression on a genome-wide scale and with specific focus on differentially regulated genes essential to cancer cell stemness, growth, survival and mitochondrial functions. Results are aligned with biological end-points and correlate with those obtained in a well-studied human GBM cell line (U87MG) [20,21,22,23] often subjected to gene expression analysis [24,25,26] and in a pancreatic cancer (SUIT2) cell line [27].

## 2. Results

### 2.1. Mechanisms of Anticancer Activities of P-bi-TAT Revealed by Genome-Wide Expression Profiling of a Primary Culture of Human GBM Cells (PC-GBM)

Present experiments identified 5689 genes expression of which was significantly altered by P-bi-TAT (1.5-fold cutoff for both 3277 up- and 2412 downregulated transcripts). A total of 250 significantly affected pathways having from 4 to 180 affected genes were identified (Supplemental Summary S1 and S2; Supplemental Table S1; both nominal and a Benjamini Hochberg [28] False Discovery Rate corrected *p* values were estimated). Mechanistically highly relevant examples of these include the following pathways: glioblastoma signaling; brain-derived neurotrophic factor (BDNF) signaling; microRNA targeting; VEGFA-VEGFR2; MAPK; EGF-EGFR; integrin-mediated adhesion; cell cycle; mitotic G1/S and G2/M phases; TNFα; DNA damage response; hepatocyte growth factor, Vitamin D and FGF receptors; JAK/STAT; IL6; IL11; IL2; IL5; TGFβ; TP53 and cell death genes; apoptosis execution phase; multiple hormonal and developmental pathways.

Strikingly, 3403 protein-coding genes (63% of all differentially regulated genes; *p* = 1.91 × 10^−52^) affected in PC-GBM cells by P-bi-TAT treatment represent human cancer survival predictor genes (Table 1; Supplemental Summary S3; Supplemental Table S2), changes in expression of which have been associated with increased likelihood of cancer patients’ survival or death after therapy [29,30,31,32]. These observations strongly suggest that the P-bi-TAT therapy preferentially targets genomic pathways sustaining the expression of human cancer survival genes. Importantly, previous genome-wide gene expression profiling experiments conclusively demonstrated highly significant associations of increased likelihood of cancer patients’ death after therapy with acquisition by cancer cells of the stemness state [32,33,34,35].

Human cancer survival predictor genes’ expression is highly significantly associated with long-term clinical outcomes in cancer patients such as 5-year survival and cancer-specific death [31]. This highly relevant clinically category of genes was identified in a pioneering study of global gene expression patterns of all protein-coding genes in the clinical samples of 33 different human cancers from 9666 patients of The Cancer Genome Atlas (TCGA) and the gene expression patterns in 37 normal human tissues obtained from 162 healthy subjects in the Human Protein Atlas (HPA) project [31]. In total, more than 100 million Kaplan–Meier plots were generated that corresponded to all 19,571 protein-coding genes across the 17 cancer types. Ultimately, these experiments identified prognostic genes for 17 different human cancer types, and they were defined as genes for which the expression level above or below the experimentally determined cutoff in an individual patient yields a significant (*p* < 0.001) difference in a patient’s survival.

There are two categories of human cancer survival predictor genes [31]. The first category represents genes for which increased expression is significantly associated with favorable prognosis (increased likelihood of survival and decreased probability of death from cancer). The second category represents genes for which increased expression is significantly associated with unfavorable prognosis (decreased likelihood of survival and increased probability of death from cancer). Therefore, the potential therapeutic implications of effects of anticancer agents on human cancer survival genes should be evaluated in the context of established correlations of patterns of the changes in their expression with patients’ survival.

Our experiments identified gene expression signatures reflecting a transcriptional reversal of death from cancer phenotypes induced by P-bi-TAT therapy: increased expression of genes associated with favorable clinical outcomes and decreased expression of genes associated with unfavorable clinical outcomes (Supplemental Summary S3). Results of these analyses are reported in Supplemental Table S2 for the 34-gene signature of the glioma cancer survival pathway. Significantly, reported findings on significant associations of the P-bi-TAT-regulated genes with survival of cancer patients are documented for changes in expressions of protein products of these genes (Supplemental Summary S3; Supplemental Table S2). Our analyses identified the 34-gene signature focused on genes previously associated with survival of the TCGA cohort of 530 glioma patients (Supplemental Table S2). Notably, cancer patients who manifested P-bi-TAT-like expression profiles had significantly better survival outcomes (Supplemental Table S2), consistent with the expected therapeutic effects of the thyrointegrin antagonists in clinical settings.

### 2.2. Examples of Specific GBM Driver Genes Important to Regulation of Cell Division, Energy Metabolism and Signal Transductions of Cancer Survival Pathways Whose Expression Is Affected by P-bi-TAT

**Figure 1:**
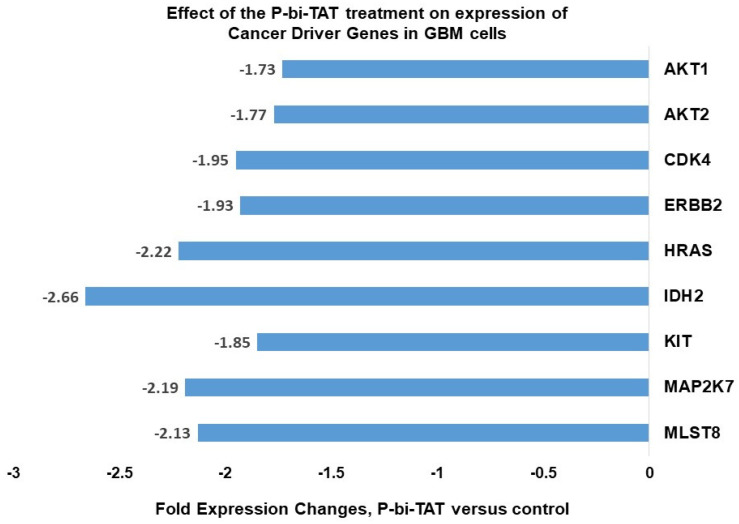
Effects of P-bi-TAT treatment on expression of selected cancer driver genes in primary GBM cells. Cancer driver genes manifesting most significant downregulation of expression in response to P-bi-TAT treatment are shown. Listed values report fold expression changes (log scale) for corresponding genes (*p* < 0.05).

The majority of cancer driver genes listed in Figure 1 whose expression is downregulated by P-bi-TAT in PC-GBM cells are involved in signal transduction that is important to cancer cell energy metabolism. These genes include *AKT1* and *AKT2*, *MAP2K7* and *MLST8*. The latter is a component of the mTOR signaling system. *HRAS* codes for a regulator of cell division but is involved in signal transduction [36]. The gene product of *IDH2* is a regulator of mitochondrial energy production [37,38], and KIT (CD117) [39], EGFR [40] and CDK4 [41] have been reported to have roles in energy metabolism in addition to other critical functions. We noted that many of these genes also have relevance to regulation of the energy metabolism in cancer cells.

### 2.3. P-bi-TAT Markedly Affects the Transcriptional Architecture of the Energy-Metabolism-Sustaining Life-Support Infrastructure of Cancer Cells

These findings prompted a more detailed follow-up exploration of the P-bi-TAT effects on expression of genes implicated in regulation and execution of metabolic processes that are essential for maintenance of the energy-producing infrastructure of cancer cells. To this end, we identified genes associated with different pathways related to the energy metabolism among all significantly affected genes and examined the numbers of up- and downregulated transcripts. Intriguingly, analyses of significantly affected genes revealed that, in all instances, expression of the majority of genes implicated in various pathways related to energy metabolism is consistently downregulated following P-bi-TAT treatment (Table 2). These observations strongly argue that one of the principal molecular components of the anticancer activity of P-bi-TAT may constitute the molecular interference with the life-sustaining energy-producing infrastructure of cancer cells. Table 2 shows a summary of the P-bi-TAT-therapy-mediated interference with energy-producing, protein synthesis and essential metabolic pathways of human primary culture GBM cells.

### 2.4. Expression of ATP Synthase Genes Is Altered by Exposure of GBM Cells to P-bi-TAT

ATP synthase is a complex of protein subunits located in the inner mitochondrial membrane and is responsible for the generation of ATP from ADP and inorganic phosphate (P_i_) [42].

**Figure 2:**
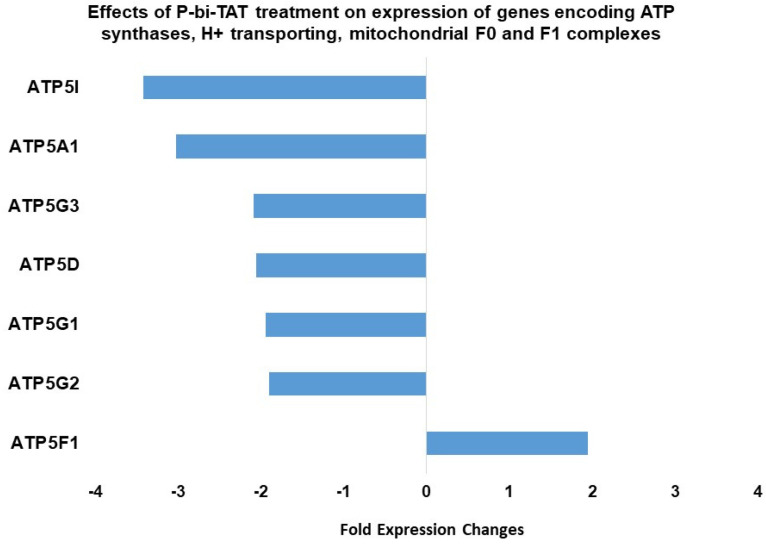
Effects of P-bi-TAT treatment on expression of genes in GBM cells encoding ATP synthases, H^+^ transporting mitochondrial F_0_ and F_1_ complexes. Listed values report fold expression changes (log scale) for corresponding genes (*p* < 0.05).

In PC-GBM cells, the expression of six ATP synthase genes whose products contribute subunit components to ATP synthase is significantly downregulated by exposure to P-bi-TAT (Figure 2), which is acting at the cell surface thyroid hormone receptor on integrin αvβ3. The genes affected contribute products to both principal subunits (F_1_, F_0_) of the synthase. The likely consequence of such a set of actions is disruption of ATP generation in mitochondria due to failure of ATP synthase assembly. We noted that a single ATP synthase gene (*ATP5F1*) related to proton channels was upregulated by P-bi-TAT, and the significance of this is not known.

### 2.5. Expression of NADH Dehydrogenase Genes in Response to Exposure of GBM Cells to P-bi-TAT

**Figure 3:**
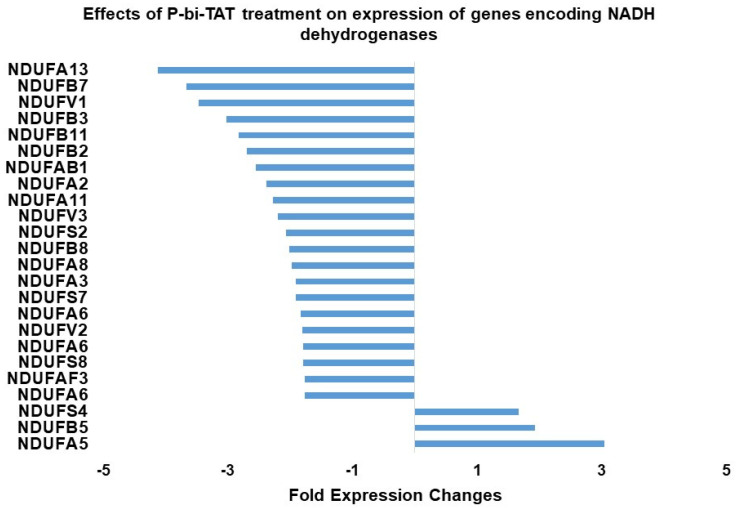
Effects of P-bi-TAT treatment on expression of genes encoding NADH dehydrogenases in primary GBM cells. Listed values report fold expression changes (log scale) for corresponding genes (*p* < 0.05). Some genes are listed several times because the expression of these genes on the array is measured by the hybridization levels of several distinct probe sets designed to capture different transcript variants of the same gene. For every significantly affected gene, all transcript variants manifesting statistically significant expression changes are reported. All listed genes manifested significant changes in expression following P-bi-TAT treatment (*p* < 0.05; see Methods).

Conversion of ADP and Pi to high-energy ATP generates protons whose disposal requires an electrochemical gradient across the inner mitochondrial membrane. A series of protein complexes functions as the electron transport chain that couples electron transfer with proton transfer. NADH dehydrogenase is a principal constituent of the electron transport chain of the inner mitochondrial membrane and consists of a large number of subunits (Complex 1 of mitochondrial redox carriers). Figure 3 shows the changes in expression of 24 NADH dehydrogenase subunit genes to P-bi-TAT. Notably, expression of 21 of these genes was significantly downregulated by P-bi-TAT, whereas expression of 3 NADH genes was upregulated by the agent. As is the case for ATP synthase genes, the action of P-bi-TAT on expression of NADH dehydrogenase subunit genes appears primarily (21 out of 24 genes) to disrupt ATP generation in the GBM cells, since expression of 21 out of 24 significantly affected genes is downregulated.

### 2.6. Concordant Patterns of Expression Changes of Energy Metabolism Genes in Human Cancer Cell Lines in Response to P-bi-TAT

**Figure 4:**
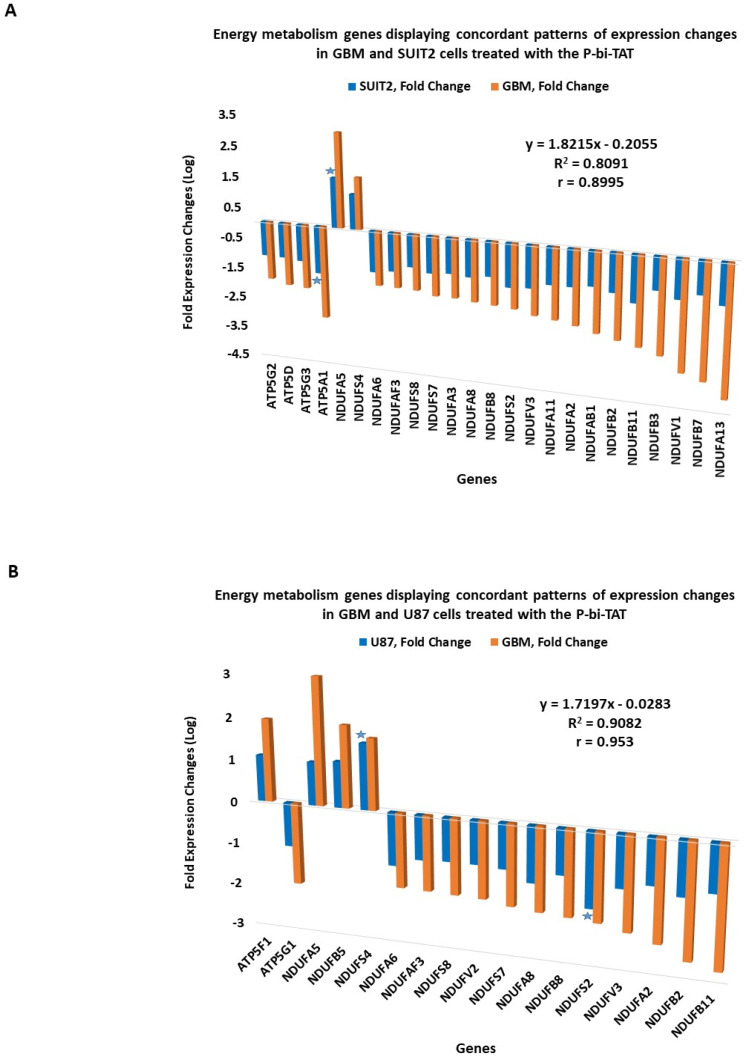
Energy metabolism genes manifesting concordant patterns of expression changes in primary culture GBM (PC-GBM) cells and established human cancer cell lines treated with the thyrointegrin inhibitor P-bi-TAT. (**A**) Pancreatic cancer SUIT2 cell line and (**B**) U87MG GBM cell line. Stars denote genes manifesting largest fold expression changes in corresponding established cancer cell lines.

It was of interest to determine whether the expression changes of energy metabolism genes observed in the GBM cells treated with the P-bi-TAT could be detected in other human cancer cell lines. To this end, we designed an expression signature of energy metabolism genes shown in Figure 2; Figure 3 and compared changes in their expression patterns in two additional human cancer cell lines (U87MG and SUIT2) treated with P-bi-TAT (Figure 4). These analyses revealed concordant patterns of P-bi-TAT-induced expression changes of energy metabolism genes in different human cancer cell lines, displaying direct correlation of gene expression changes between the PC-GBM and SUIT2 cells (r = 0.8995, Figure 4A) and the PC-GBM and U87MGGBM cells (r = 0.953, Figure 4B). Of note, the magnitude of P-bi-TAT-induced gene expression changes in established cancer cell lines cultured on plastic seems less significant than in primary GBM cells. These findings suggest that expression changes of energy metabolism genes may represent one of the common mechanisms of anticancer activities of the P-bi-TAT and other molecules representing tetrac-based therapeutics.

### 2.7. Concordance of Biological Activities and Molecular Mechanisms of Actions of P-bi-TAT

Marked anticancer activity of P-bi-TAT was recently demonstrated for this novel anticancer agent in both in vitro and in vivo experiments [7]. However, mechanisms of its anticancer activity remain to be fully elucidated. Previous peer-reviewed studies from our laboratories, which were dedicated to the discovery and in-depth preclinical investigations of both endogenous and synthetic thyrointegrin αvβ3 antagonists, have demonstrated that one of the major molecular mechanisms of their nongenomic bioactivity against target cells is captured by their global multifaceted effects on gene expression [2,3,4,5,6,43,44]. This concept is best illustrated by the molecular interference model with multiple intracellular signal transduction pathways essential for growth and survival of cancer cells. The relevance of the corresponding in vitro models as molecular surrogates of the in vivo activity of thyrointegrin αvβ3 antagonists has been documented in the experiments reported in multiple peer-reviewed publications [2,3,6,43,44].

Therefore, it was logical to employ this experimental strategy with regard to the novel, more potent thyrointegrin αvβ3 antagonist P-bi-TAT in order to dissect the potential molecular mechanisms of its anticancer and anti-angiogenic activities. Results of experiments reported in this paper demonstrate that P-bi-TAT acts similarly to other thyrointegrin αvβ3 antagonists. The major biological determinants of anticancer activity of P-bi-TAT are highlighted by its anti-angiogenic, pro-apoptotic and anti-proliferative activities (Table 3; Supplemental Summary S1 and S2; Supplemental Table S1). Specifically, we observed that in both GBM and metastatic pancreatic cancer models of human malignancies, the most significantly affected pathway is the VEGFA-VEGFR2 signaling pathway (Table 3). These findings are highly consistent with the experimental observations documenting marked anti-angiogenic activity in vitro and in vivo of the P-bi-TAT and other thyrointegrin αvβ3 antagonists [2,7,43,44,45]. In addition, present observations provide additional evidence advancing further the molecular interference model of nongenomic bioactivity of the thyrointegrin αvβ3 antagonists by describing the in-depth molecular anatomy of gene expression changes affecting more than 200 signal transduction pathways that are the most significantly influenced by P-bi-TAT treatment (Table 3; Supplemental Summary S1 and S2; Supplemental Table S1).

**Figure 5:**
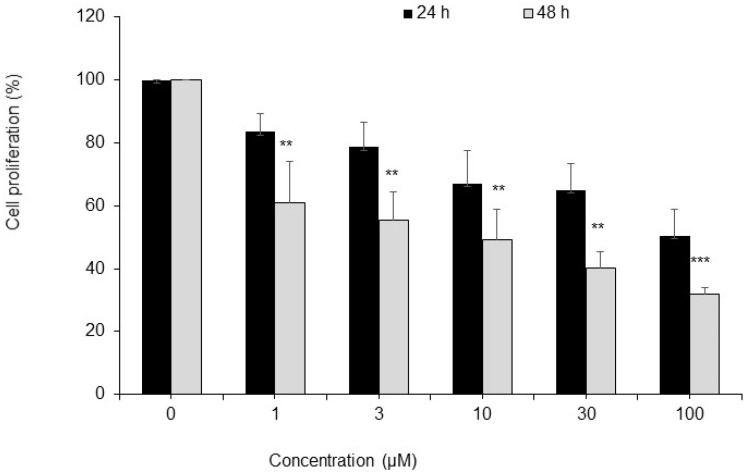
Cell proliferation assay demonstrates dose- and time-dependent inhibitory effects of the P-bi-TAT on human glioblastoma cells. U87-luc cells were incubated with P-bi-TAT at different concentrations (1, 3, 10, 30 and 100 µM) for 24 h and 48 h and were measured with MTT assay. Values are presented as mean ± SD of three independent experiments. ** *p* < 0.01, *** *p* < 0.001, compared to control (PBS).

**Figure 6:**
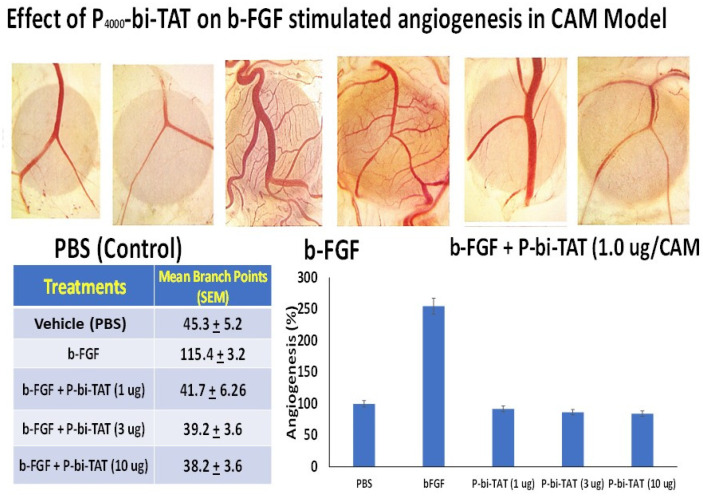
Angiogenesis assay results from chick chorioallantoic membrane (CAM) model demonstrate the inhibitory effect of P-bi-TAT on blood vessel formation induced by bFGF. Top panel of images shows representative CAM angiogenesis fields (in duplicates) of the PBS control, bFGF-induced blood vessel growth and lack of bFGF-induced blood vessel growth in the presence of P-bi-TAT. Bottom images report the inhibitory effect of the P-bi-TAT on bFGF-induced angiogenesis documented in a tabular format (Mean ± SEM) and the quantitative visualization of results as bar graphs. Pro-angiogenic bFGF concentration was 1 μg/mL in PBS.

To corroborate results of analyses of molecular mechanisms of P-bi-TAT actions, we evaluated biological effects of the P-bi-TAT on cancer cell proliferation and angiogenesis. MTT cell proliferation assay demonstrates dose- and time-dependent inhibitory effects of the P-bi-TAT on proliferation of U87-luc human glioblastoma cells (Figure 5). Using the chick chorioallantoic membrane (CAM) model [11,12], of angiogenesis in ovo, we evaluated the effects of P-bi-TAT on growth of blood vessels. These experiments documented potent inhibitory effects of the P-bi-TAT on angiogenesis (Figure 6). The potent anti-angiogenic activity of P-bi-TAT was in accord with previously reported actions of other thyrointegrin antagonists [12].

### 2.8. Naïve Pluripotency Network Genes of Human Preimplantation Embryo Comprise a Marked Majority of the P-bi-TAT Target Genes in Human GBM Cells

**Figure 7:**
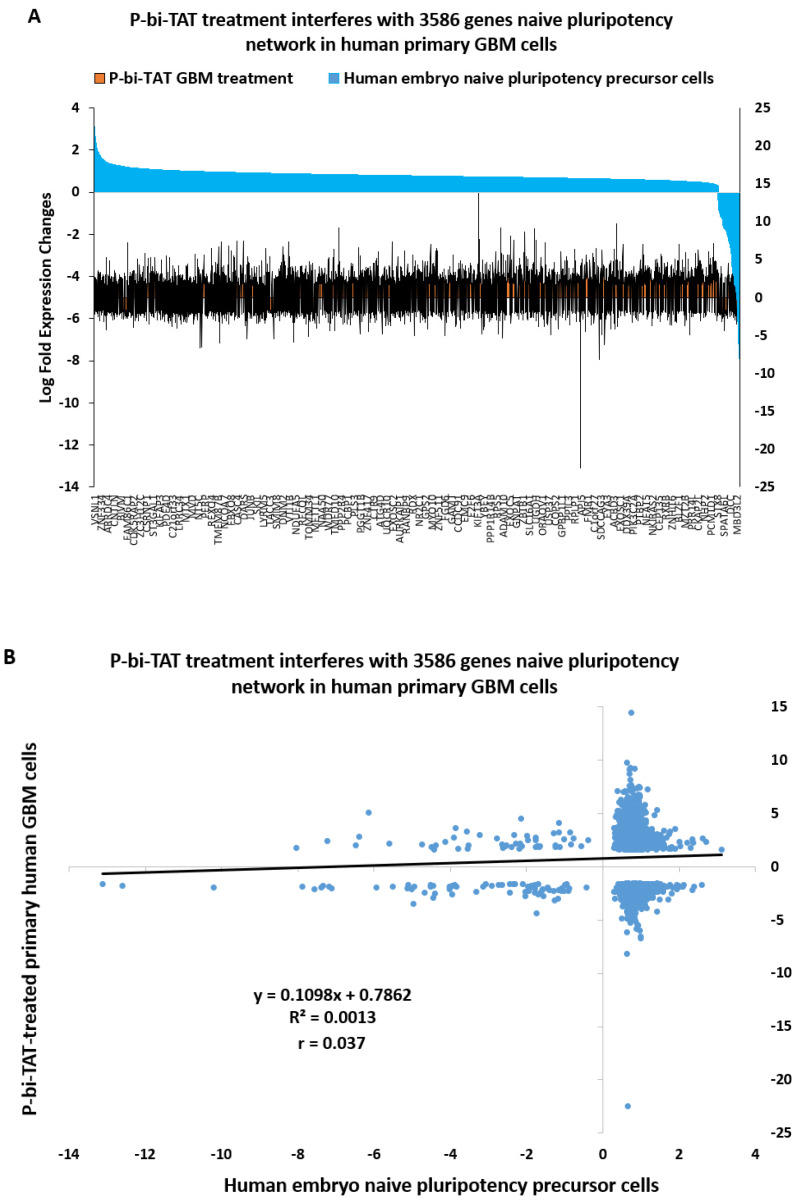
Treatment of human GBM cells with the P-bi-TAT thyrointegrin antagonist exerts disorderly effects on expression of 3586 genes of naïve pluripotency networks operating in malignant cells. The disorderly effects of the P-bi-TAT on stemness pathways’ gene expression are documented by analyses of gene expression profiles (**A**) and correlation patterns (**B**). See Text and Supplemental Table S3 for details.

We noted that among significantly affected pathways of potential biological relevance, there were multiple genes and pathways associated with stemness phenotype, suggesting stem cell signal transduction pathways operating in human GBM cells may be affected by the P-bi-TAT treatment. To explore this hypothesis further, we investigated whether the P-bi-TAT treatment interferes with gene expression of the naïve pluripotency transcriptional network operating in multi-lineage markers expressing (MLME) cells of human preimplantation embryos [32,33,34,35,46,47,48,49]. Our analysis identified 3586 genes (63% of all P-bi-TAT differentially regulated genes; *p* = 0.000; two-tail Fisher’s exact test) of the naïve pluripotency network of human preimplantation embryos, expression of which is significantly affected by the P-bi-TAT treatment (Figure 7).

In total, there were 2228 upregulated and 1358 downregulated stemness network genes affected by the P-bi-TAT treatment (Figure 7; Supplemental Table S3), comprising 68% and 56% of all P-bi-TAT target genes in corresponding categories (Supplemental Table S3). Notably, highly ordered expression profiles of genes comprising naïve pluripotency transcriptional network of the MLME cells of human preimplantation embryos appear markedly distorted by the P-bi-TAT treatment (Figure 7). Collectively, these findings are consistent with the hypothesis that P-bi-TAT therapy interferes with functions of stemness signaling pathways operating in human GBM cells. Gene set enrichment analyses (GSEA) of 3586 naïve pluripotency network genes revealed significantly affected signaling pathways of potential mechanistic relevance highlighting biological functions of GBM cells that might be affected by the P-bi-TAT treatment (Supplemental Table S3). Taken together with observations reported above, these findings indicate that signal transduction pathways regulating energy metabolism and survival of human GBM cells may represent intrinsic components of stemness genomic networks operating in malignant cells.

## 3. Discussion

Preclinical development and clinical validation of novel therapeutic modalities for efficient treatment of malignant brain tumors represent one of the highly significant challenges of contemporary experimental and clinical oncology [50,51,52,53]. Experimental therapeutics of tetrac and its formulations have shown promising in vitro and in vivo activities against a broad spectrum of human malignancies, including gliomas [11,14,43,44,54]. Changes in gene expression in human cancer cells induced by the exposure to tetrac and Nanotetrac (the nanoparticulate PLGA formulation of tetrac) have been shown to occur prior to any detectable changes in either growth or viability of cancer cells, suggesting that effects on gene expression represent a key mechanistic determinant of anticancer activity of the tetrac family of therapeutics [1,2,4,43,55,56].

**Figure 8:**
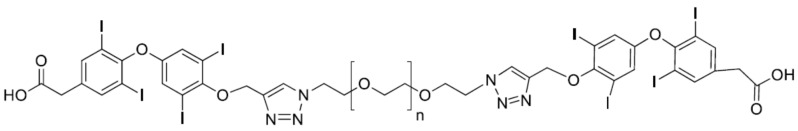
Molecular structure of the P-bi-TAT as reported in our previously published study [7]. Adopted from our previously published contribution [7]. Copyright © 2022 American Chemical Society.

Recently, we reported synthesis of novel thyrointegrin receptor antagonist P-bi-TAT (Figure 8) and documented its potent anti-cancer activity in vivo in human xenograft mouse models of GBM [7]. To gain insight into mechanisms of anticancer activity of P-bi-TAT, we carried out genome-wide expression profiling analyses of primary human glioblastoma cells treated with nontoxic doses of P-bi-TAT and compared the results with those from a standard GBM cell line and a pancreatic cancer cell line treated with P-bi-TAT. Because of the recent preclinical description of the effectiveness of drug-targeting of mitochondrial ATP synthase activity in GBM cells [9], we also explored the possible activity of P-bi-TAT on expression of ATP synthase and NADH dehydrogenase (see analysis below).

Observations reported here indicate that the P-bi-TAT therapy preferentially targets genetic pathways sustaining the expression of human cancer stemness and survival genes. Among the genes showing expression both affected by P-bi-TAT treatment and significantly associated with the survival of glioma patients, we identified a 34-gene signature reflecting a transcriptional reversal of cancer cells’ survival or death from cancer phenotypes: increased expression of genes associated with favorable clinical outcomes, and decreased expression of genes associated with unfavorable clinical outcomes. P-bi-TAT administration significantly affects expression of hundreds of genes associated with many therapeutically important pathways, including key biological pathways linked to tetrac and its nanoparticle formulations. Multiple pathways were affected in GBM cells, and the targeting of these cells by tetrac-based therapeutics was previously validated using multiple independent models based on both biological and analytical assays [2,14,57].

Collectively, these results support the mechanistic model of the P-bi-TAT action being initiated at an integrin cell surface receptor to interfere with tumor cell proliferation and tumor-associated angiogenesis, as well as to disrupt functions of apoptosis resistance and DNA repair pathways. Overall, we observed the changes in gene expression in response to the P-bi-TAT treatment that are consistent with the molecular interference model. Genes that are downregulated in stem cells are upregulated following the P-bi-TAT treatment. Conversely, genes that are upregulated in stem cells manifest decreased expression after the P-bi-TAT treatment. Similar patterns were observed for gene expression signatures induced in cancer cells following exposures to various growth factors and other endogenous ligands: genes that are upregulated in cancer cells in response to growth factor exposure are downregulated following the P-bi-TAT treatment, while genes expression of which is inhibited in cancer cells in response to growth factors manifest increased expression after the P-bi-TAT treatment.

We have recently shown that P-bi-TAT significantly reduces the size of human GBM xenografts [7], with persistence of the anticancer effect after interruption of drug therapy. The drug administration protocol in the current study was designed to achieve a circulating P-bi-TAT concentration comparable to that evaluated in our 2019 study. In earlier evaluations of a pharmaceutical containing a single tetrac (nano-diamino-tetrac, Nanotetrac), rather than the two tetrac molecules of P-bi-TAT, we found actions on the expression of certain driver genes and genes linked to angiogenesis, regulation of the cell cycle, cancer cell survival pathways in breast cancer cells [2,10,43] and medullary carcinoma of the thyroid cells [10]. The results of experiments reported in the present study of P-bi-TAT are also consistent with the concept that changes in gene expression in human cancer cells represent a principal mechanistic determinant of the anticancer activity of tetrac-containing agents [10,11,12,13]. Because the access of Nanotetrac and P-bi-TAT to the nuclear compartment of cancer cells is severely restricted [1,3], these observations suggest that the principal molecular target of P-bi-TAT in the present studies is the thyroid hormone analogue receptor on integrin αvβ3.

The present study indicates that expression of a substantial number of GBM genes can be differentially regulated at the integrin αvβ3 receptor and that these genes are highly relevant to control of cancer cell respiration. Among these genes are a number that are important to electron transport in mitochondria and thus to the generation of ATP. Chemically modified tetrac can disrupt the networks of ATP synthases and NADH dehydrogenases in the inner mitochondrial membrane that are essential to cancer cell metabolism, as documented in this report. A small molecule, gboxin, which disrupts the activity of mitochondrial ATP synthase, has been shown by others to be an effective experimental anti-GBM drug [9]. It is understandable that reduction or elimination of ATP will affect viability of targeted cells. However, in the case of tetrac-containing drugs, which are capable of inducing tumor cell death by apoptosis [4,57] or by other ATP-dependent mechanisms [58], the availability of ATP may determine whether apoptosis can be induced [59,60]; the drug itself may be determining ATP availability. Studied in GBM cells in vitro, ATP-dependent apoptosis caused by tetrac-containing agents is not apparent for 2–3 days [44]. Thus, actions of drugs on ATP synthases to substantially reduce ATP production in cancer cells may not impair apoptosis induction for several days. This may become a consideration when ATP-synthase-inhibiting agents and apoptosis-inducing drugs are combined in therapy.

The modest expression of αvβ3 in normal cells means that the actions of modified tetrac molecules—such as P-bi-TAT—have little or no effect on noncancer cells, including nondividing endothelial cells. Thus it is unlikely that ATP generation in normal cells is affected by modified tetrac. We have examined the systemic effects of another tetrac formulation (Nanotetrac) in tumor-xenograft-bearing nude mice and have not found systemic effects on normal cells and tissues [10,13,44].

Another implication of the current results is that T4, the natural ligand of the thyroid hormone αvβ3 (thyrointegrin) receptor, may support metabolism in tumor cells and actively angiogenic blood vessel cells that serve tumors [4]. Thus, certain of the distinctive metabolic characteristics of cancer cells may not be intrinsic but conferred by circulating T4 at αvβ3 on the cell surface. Cellular abundance of ATP may in part be determined from the plasma membrane in tumor cells and blood vessel cells in cancers. The striking dichotomy of actions on tumor cells of the endogenous thyroid hormone analogue inventory consisting of T4/tetrac/T3 may be driven by the distinct affinities toward intracellular/nuclear (T3 >> T4 > tetrac) versus extracellular/cell membrane (tetrac > T4 >> T3) receptors [4]. Furthermore, the distinct biological effects of tetrac and T4 on cancer cells, but not on normal cells, might be a function of the accessibility of the cell surface thyroid hormone analogue receptors. In cohesive tissues of the human body, cells are tightly bound to each other and to the extracellular matrix via cadherins, integrins, tight gaps and adherent junctions. These interactions form networks of anchoring, communication and occluding junctions, which are essential for physiological functions of coherent tissues and organs but are demolished in malignant tumors. Consequently, the accessibility to cell surface receptors on normal versus malignant cells for T4 and tetrac would be substantially different: the access to receptors would be restricted on normal cells and readily available on cancer cells with diminished adhesion properties. The validity of these models and delineation of molecular distinctions among these possibilities certainly require further investigation.

## 4. Materials and Methods

### 4.1. P-bi-TAT

P-bi-TAT was synthesized in the authors’ laboratory by the method of M. Rajabi et al. [7].

### 4.2. Cells and Cell Culture Conditions; Treatment with P-bi-TAT

A primary culture of human GBM cells (PC-GBM; GBM 021,913, kindly provided by the Department of Neurosurgery, University of Pittsburgh Medical Center, Pittsburgh, PA, USA) and a human GBM cell line (U87MG, obtained from ATCC, Manassas, VA, USA) were studied. These were cultured in RPMI medium containing 10% FBS at 37 °C and 5% CO_2_/95% air. For 2 days prior to cell exposure to P-bi-TAT, cells were maintained in medium containing 0.25% FBS and then exposed to 30 µM P-bi-TAT for 24 h. Control cells were exposed to PBS, pH 7.4, for the same period of time. Additional experiments were carried out on the human pancreatic cancer (SUIT2) cell line, obtained and cultured as previously described [27].

### 4.3. Cell Proliferation Assay

Glioblastoma cells (U87-luc) were seeded in 96-well plates (0.5 million cells per well) and treated with the P-bi-TAT at five concentrations (1; 3; 10; 30; and 100 μM). At the end of the experiments (24 and 48 h), cell cultures were supplemented with MTT reagent and incubated for an additional 4 h. Then, DMSO was added to the cell culture and incubated for 10 min at room temperature to dissolve the formazan crystals. The absorbance rate of the processed cell cultures was read at 570 nm using a microplate reader. All reactions were carried out in biological triplicates. Data estimates of cell proliferation were calculated using the values of PBS-treated control cells.

### 4.4. Analysis of the P-bi-TAT Effects on Angiogenesis

Angiogenesis assay was performed using the chick chorioallantoic membrane (CAM) model of angiogenesis in ovo as previously described [11,12]. We evaluated the effects of P-bi-TAT on blood vessel formation induced by basic fibroblast growth factor (bFGF; also known as FGF2) at 1 μg/mL. In brief, neovascularization was examined in the CAM model using 10-day-old chick embryos purchased from Charles River Avian Vaccine Services (Norwich, CT, USA) and incubated at 37 °C with 55% relative humidity. A hypodermic needle was used to make a small hole in the shells at the air sacs, and a second hole was made on the broadside of the eggs, directly over an avascular portion of the embryonic membrane that was identified by candling. A false air sac was created beneath the second hole by the application of negative pressure at the first hole, causing the CAM to separate. A window of approximately 1.0 cm^2^ was cut in the shell over the dropped CAM using a small craft grinding wheel (Dermal, Division of Emerson Electric Co. Racine, WI, USA), allowing for direct access to the underlying membrane. bFGF was used as a standard proangiogenic agent. To deliver the P-bi-TAT, sterile disks of No. 1 filter paper (Whatman International, Kent, UK) were pretreated with P-biTAT, air-dried under sterile conditions and placed on the CAMs. After incubation at 37 °C with 55% relative humidity for 3 days, CAM tissues directly beneath each filter disk were resected, washed with PBS, placed in 35 mm Petri dishes (Nalge Nunc, Rochester, NY, USA) and examined under SV6 stereomicroscope (Carl Zeiss, Thornwood, NY, USA) at ×50 magnification. Digital images of CAM sections (control and exposed to the treatment) were collected using a 3-CCD color video camera system (Toshiba America, New York, NY, USA) and analyzed with Image-Pro software (Media Cybernetics, Silver Spring, MD, USA). The number of vessel branch points contained in a circular region selected to be equal to the area of each filter disk was counted. A single image was acquired for each CAM preparation, and 8 CAM preparations were processed per control and treatment conditions. Results are reported as the mean ± SD of the numbers of new branch points.

### 4.5. Microarray

Total RNA was harvested isolated using Trizol and checked for quality using a Bioanalyzer before being used for microarray analysis. RNAs were processed for hybridization into the Clariom™ S human assay platform (Affymetrix, Santa Clara, CA, USA) at the Center for Functional Genomics, University at Albany, Rensselaer, NY. Briefly, total RNA (100 ng) was processed using the WT Plus Reagent kit (Affymetrix). Sense target cDNAs were generated using the standard Affymetrix WT protocol and hybridized to Affymetrix Human Clariom S arrays. Arrays were washed, stained and scanned on a GeneChip 3000 7G scanner using Affymetrix GeneChip Command Console Software (AGCC). Transcriptome Analysis Console Software (TAC v3.0.1.5) was used to identify differentially expressed genes. The CEL files were summarized using the SST-RMA algorithm in TAC, and the normalized data were subjected to one-way ANOVA with a Benjamini Hochberg False Discovery Rate correction included (*p* < 0.05) [28]. A 1.5-fold change was used to select entities that were statistically and differentially expressed between the conditions being compared (treated and untreated). Gene set enrichment analyses (GSEA) of genes’ expression that was significantly affected by P-bi-TAT treatment identified a total of 250 significantly affected pathways having from 4 to 180 affected genes in the GBM cells and 39 significantly affected pathways having 4 to 29 genes in the SUIT2 cells (Supplementary Summary S1 and S2).

The workflow of microarray analyses was reported in previously published contributions [29,30,32,43]. Gene set enrichment analyses (GSEA) of differentially expressed genes (DEGs) were carried out using the Enrichr bioinformatics platform, which enables the interrogation of nearly 200,000 gene sets from more than 100 gene set libraries. The Enrichr API (January 2018 through June 2021 releases) [61,62,63] was used to test DEGs of interest for significant enrichment. Different sets of DEGs defined at multiple significance levels of statistical metrics and comprising from dozens to several thousand individual genetic loci were analyzed using differential GSEA to identify pathways associated with biological effects of DEGs and infer potential mechanisms of P-bi-TAT anticancer activities. Analyses of large numbers of DEGs often comprising gene expression signatures of numerous pathways represent a formidable challenge, which is often not satisfactorily addressed by automated computational approaches. To this end, the differential GSEA approach combined with genome-wide proximity placement analysis (GWPPA) was developed and successfully implemented for identification and characterization of human-specific regulatory networks governed by human-specific transcription factor-binding sites [33,34,46,64,65], more than 24,000 genes linked with hESC functional enhancer elements [47,48,49], 13,824 genes associated with 59,732 human-specific regulatory sequences [66], 8405 genes associated with 35,074 human-specific neuroregulatory single-nucleotide changes [67] and 8384 genes regulated by stem cell-associated retroviral regulatory sequences [35]. Initial GSEA entail interrogations of each specific set of DEGs (all statistically significant DEGs; upregulated DEGs; downregulated DEGs) using ~30 distinct genomics and proteomics databases, including pathway enrichment Gene Ontology (GO) analyses followed by in-depth interrogations of most informative gene subsets and the selected genomic databases deemed most statistically and biologically relevant. In all reported tables and figures related to GSEA, in addition to the nominal *p* values and adjusted *p* values (corrected for multiple hypothesis testing), the statistical metrics designated “combined scores” were calculated by the Enrichr software [61,62,63], which represent a product of the significance estimate and the magnitude of enrichment (combined score c = log(p) * z, where *p* is the Fisher’s exact test *p*-value, and z is the z-score deviation from the expected rank).

Microarray raw data were deposited in GEO under the series GSE140272 and GSE140449.

## 5. Conclusions

Reported herein observations are based on experiments employing synthetic molecules designed to act as thyrointegrin antagonists targeting thyroid hormone receptors on outer cell membranes with highly restricted access to cells’ interior. Therefore, documented molecular interference effects on signal transduction pathways sustaining cancer cells’ stemness state and energy metabolism networks are mechanistically linked with altered functions of cells’ membrane. Mechanistically, targeted engagement and retention of nano-scale drug molecules on cells’ membrane would impede the lateral mobility of macromolecules and interfere with the assembly of multimolecular complexes mediating signal transduction events into cells’ interior. The following conclusions were possible to reach using the thyrointegrin antagonists’ experimental approach:

1. Energy metabolism gene expression pathways represent an intrinsic component of stemness and cancer survival networks engaged in malignant cells;
2. The association between cancer cells’ stemness state, survival networks and energy metabolism pathways revealed by thyrointegrin antagonist actions was observed in primary GBM cells and appears less evident in cancer cells adapted to in vitro cell culture conditions;
3. This apparent dichotomy likely reflects different states of cancer cells’ adaptations to strikingly distinct in vivo and in vitro microenvironmental conditions favoring deployments of different energy metabolism mechanisms. Some important examples of such microenvironmental factors include availability of nutrients, degrees of oxygen accessibility and milieu acidification reaching extreme hypoxic and acidic conditions in vivo;
4. In addition to fundamental and mechanistic considerations, documented effects of P-bi-TAT on gene expression of cancer stemness, survival and energy metabolism networks highlight a powerful therapeutic interference opportunity with growth and survival of malignant cells.

## Figures and Tables

**Table 1:** P-bi-TAT treatment alters expression of a large number of human cancer survival genes. *p* values were estimated using the hypergeometric distribution test.

Classification Category	Number of Differentially Regulated Genes	Number of Cancer Survival Genes	Percent of Cancer Survival Genes	*p* Value
**Pancreatic cancer (SUIT2)**	1293	860	67	1.47 × 10^−19^
**Top upregulated (*p* = 0.001)**	50	40	80	0.000124
**Top downregulated (*p* = 0.001)**	15	12	80	0.030497
**Top differentially regulated (*p* = 0.001)**	65	52	80	1.32 × 10^−5^
**Glioblastoma multiforme (GBM)**	5362	3403	63	1.91 × 10^−52^
**Top upregulated (6-fold)**	66	47	71	0.002467
**Top downregulated (2.5-fold)**	106	64	60	0.039735
**Top differentially regulated**	172	111	65	0.002074
**Consensus (SUIT2 and GBM)**	737	501	68	2.5 × 10^−14^
**Top upregulated (4.9-fold)**	68	57	84	2.91 × 10^−7^
**Top downregulated (2.5-fold)**	61	43	70	0.004617
**Top differentially regulated**	129	100	78	3.88 × 10^−8^

Legend: *p* values were estimated using the hypergeometric distribution test.

**Table 2:** Summary of the P-bi-TAT-therapy-mediated interference with energy-producing, protein synthesis and essential metabolic pathways of human primary culture glioblastoma multiforme (GBM) cells.

Classification Category	Downregulated Genes
**Electron transport chain**	*ATP5A1, ATP5I, COX6B1, ATP5G2, NDUFA8, NDUFA3, NDUFV2, NDUFA6, NDUFA2, COX5A, NDUFS7, COX6A1, COX4I1, SLC25A6, NDUFB3, ATP5G1, COX7A2, ND6, NDUFAB1, COX7B, NDUFB7, UQCRC1, COX5B, COX8A, NDUFV1, ATP5G3, SURF1, NDUFB2, NDUFS2, ATP5D, NDUFV3, NDUFA10, UCP2, NDUFS8, NDUFB8*
**Cytoplasmic ribosomal proteins**	*RPL10A, RPL8, RPL9, RPLP2, RPLP1, RPL35, RPL7A, RPL13, RPL14, RPL18A, RPL18, RPL19, RPL21, RPL27, RPL28, RPL29, RPL32, RPL39, UBA52, RPL41, RPL36A, RPS3, RPS9, RPS5, RPS15A, RPS16, RPS20, RPS14, RPS29, RPS11, RPS15, RPS7, RPS8, RPS10, RPS19, RPS26, RPS27, RPS27A, RPS28, FAU, RPLP0, RPS6KA1, RPL11, RPL10, RPL30, RPS2, RPS6KB2*
**Oxidative phosphorylation**	*ATP5A1, ATP5D, ATP5G2, ATP5G1, ATP5G3, ATP5I, NDUFA11, NDUFS7, NDUFA2, ND6, NDUFA8, NDUFS2, NDUFS8, NDUFB2, NDUFV2, NDUFV3*
**Metabolism of carbohydrates**	*SLC25A1, PCK1, SLC25A10, GALK1, GALT, PGLS, SLC37A4, AKR1B1, AKR1A1*
**Glucose metabolism**	*SLC25A1, PCK1, SLC25A10, SLC37A4*
**Fatty acyl-CoA and cholesterol biosynthesis**	*SLC25A1, PPT2, SLC27A3, FDPS, MVD, DHCR7, PMVK, FDFT1, MVK*
**Globo sphingolipid metabolism**	*ST3GAL1, ST6GALNAC4, ST6GALNAC6, ST6GAL1*
**Biogenic amine synthesis**	*DDC, ACHE, COMT*

**Table 3:** Effects of P-bi-TAT treatment on gene expression of sixteen common pathways significantly affected in both human glioblastoma multiforme (GBM) and metastatic pancreatic carcinoma (SUIT2) cells. Genes’ expression that was significantly affected by P-bi-TAT treatment was independently identified in GBM and SUIT2 cells and subjected to gene set enrichment analyses to identify significantly enriched pathways. Affected genes are listed in Supplemental Table S1.

Pathway	Cancer Model	Number of Genes	*p*-Value	Cancer Model	Number of Genes	*p*-Value
**VEGFA-VEGFR2 signaling pathway**	GBM	85	0.011056	SUIT2	29	0.00245
**Androgen receptor signaling pathway**	GBM	43	0.000046	SUIT2	13	0.00843
**Brain-derived neurotrophic factor (BDNF) signaling pathway**	GBM	54	0.020347	SUIT2	19	0.00678
**Deubiquitination**	GBM	13	0	SUIT2	4	0
**Endoderm differentiation**	GBM	53	0.025638	SUIT2	17	0.02808
**Focal adhesion**	GBM	69	0.011855	SUIT2	24	0.00308
**Gastric cancer network 2**	GBM	15	0.027934	SUIT2	7	0.00467
**Human thyroid-stimulating hormone (TSH) signaling pathway**	GBM	30	0.002383	SUIT2	10	0.01235
**IL-6 signaling pathway**	GBM	22	0.001737	SUIT2	9	0.00193
**Integrin-mediated cell adhesion**	GBM	40	0.013987	SUIT2	14	0.0085
**Interleukin-11 signaling pathway**	GBM	27	0.000004	SUIT2	8	0.00831
**MAPK signaling pathway**	GBM	16	0.002772	SUIT2	21	0.00768
**Olfactory receptor activity**	GBM	47	0	SUIT2	6	4 × 10^−6^
**Signaling of hepatocyte growth factor (HGF) receptor**	GBM	16	0.020177	SUIT2	6	0.02432
**TCF-dependent signaling in response to WNT**	GBM	14	0	SUIT2	9	0.00539
**TGF-beta signaling pathway**	GBM	61	0.000019	SUIT2	17	0.01388

## Data Availability

The microarray raw data presented in this study are openly available in GEO series, accession number: GSE140272, GSE140449.

## Supplementary Materials

The following supporting information can be downloaded at: https://www.mdpi.com/article/10.3390/metabo12040325/s1, Supplementary Summary S1: P-bi-TAT effects on the genetic base of the life support infrastructure of glioblastoma multiforme, Supplementary Summary S2: P-bi-TAT effects on gene expression, Supplementary Summary S3: P-bi-TAT cancer survival pathways, Table S1: GBM and PancCa common pathways, Table S2: Thirty-four glioma survival genes, Table S3: P-bi-TAT stemness.
