# Supplementary material for: Effects of Anticancer Agent P-bi-TAT on Gene Expression Link the Integrin Thyroid Hormone Receptor to Expression of Stemness and Energy Metabolism Genes in Cancer Cells
Source: Metabolites. 2022 Apr 4;12(4):325. doi: 10.3390/metabo12040325 (PMC9029602; doi:10.3390/metabo12040325)
Supplement: Supplementary file 1 [file metabolites-12-00325-s001.zip › Supp Summary S1. P_bi_TAT effects on the genetic base of the life support infrastructure of Glioblasoma Multiforme.pdf]

P-bi-TAT destroys the transcriptional architecture of the life-support infrastructure networks of cancer cells

STUDY SUMMARY

Effect of the P-bi-TAT treatment on gene expression

Cell line	Number of significantly affected genes	Up-regulated	Down-regulated	Number of significantly affected pathways	Number of affected genes in pathways
SUIT2-luc	1348	825	523	39	4 - 29
GBM 021913	5689	3277	2412	250	4 - 180

Filter criteria:

Fold change >1.5 or < -1.5

P - Value < 0.05

P-bi-TAT destroys the transcriptional architecture of the life-support infrastructure of cancer cells

DECREASED EXPRESSION OF GENES REGULATING DNA SYNTHESIS, REPLICATION, AND CELL CYCLE PROGRESSION

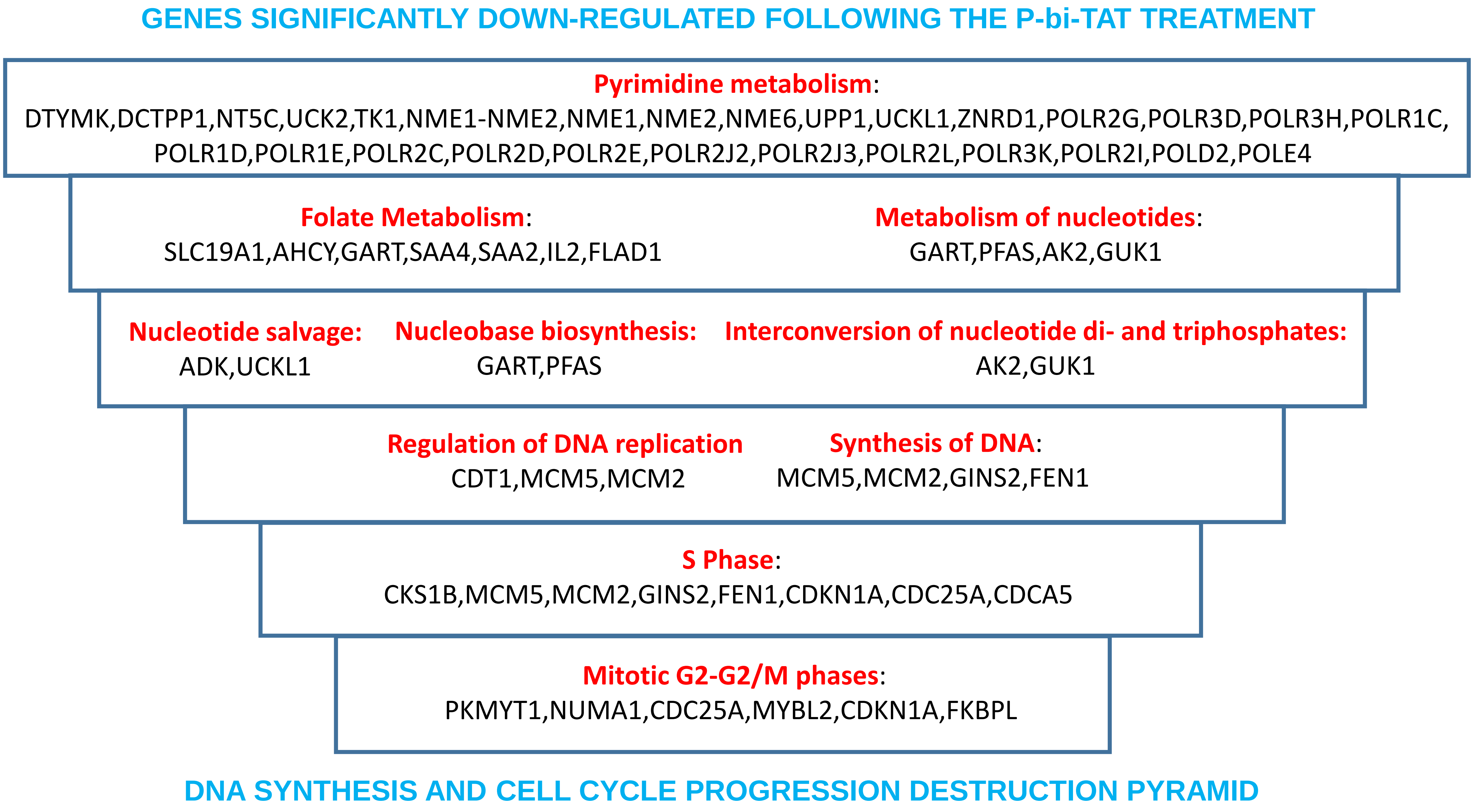
GENES SIGNIFICANTLY DOWN-REGULATED FOLLOWING THE P-bi-TAT TREATMENT
Pyrimidine metabolism:
DTYMK,DCTPP1,NT5C,UCK2,TK1,NME1-NME2,NME1,NME2,NME6,UPP1,UCKL1,ZNRD1,POLR2G,POLR3D,POLR3H,POLR1C,
POLR1D,POLR1E,POLR2C,POLR2D,POLR2E,POLR2J2,POLR2J3,POLR2L,POLR3K,POLR2I,POLD2,POLE4
Folate Metabolism:
SLC19A1,AHCY,GART,SAA4,SAA2,IL2,FLAD1
Metabolism of nucleotides:
GART,PFAS,AK2,GUK1
Nucleotide salvage:
ADK,UCKL1
Nucleobase biosynthesis:
GART,PFAS
Interconversion of nucleotide di- and triphosphates:
AK2,GUK1
Regulation of DNA replication
CDT1,MCM5,MCM2
Synthesis of DNA:
MCM5,MCM2,GINS2,FEN1
S Phase:
CKS1B,MCM5,MCM2,GINS2,FEN1,CDKN1A,CDC25A,CDCA5
Mitotic G2-G2/M phases:
PKMYT1,NUMA1,CDC25A,MYBL2,CDKN1A,FKBPL
DNA SYNTHESIS AND CELL CYCLE PROGRESSION DESTRUCTION PYRAMID

P-bi-TAT destroys the transcriptional architecture of the life-support infrastructure of cancer cells

Interference with energy-producing, protein synthesis, and essential metabolic pathways

GENES SIGNIFICANTLY DOWN-REGULATED FOLLOWING THE P-bi-TAT TREATMENT

Electron Transport Chain:
ATP5A1,ATP5I,COX6B1,ATP5G2,NDUFA8,NDUFA3,NDUFV2,NDUFA6,NDUFA2,COX5A,NDUFS7,COX6A1,COX4I1,SLC25A6, NDUFB3,ATP5G1,COX7A2,ND6,NDUFAB1,COX7B,NDUFB7,UQCRC1,COX5B,COX8A,NDUFV1,ATP5G3,SURF1,NDUFB2, NDUFS2,ATP5D,NDUFV3,NDUFA10,UCP2,NDUFS8,NDUFB8

Cytoplasmic Ribosomal Proteins:
RPL10A,RPL8,RPL9,RPLP2,RPLP1,RPL35,RPL7A,RPL13,RPL14,RPL18A,RPL18,RPL19,RPL21,RPL27,RPL28,RPL29,RPL32, RPL39,UBA52,RPL41,RPL36A,RPS3,RPS9,RPS5,RPS15A,RPS16,RPS20,RPS14,RPS29,RPS11,RPS15,RPS7,RPS8,RPS10, RPS19,RPS26,RPS27,RPS27A,RPS28,FAU,RPLP0,RPS6KA1,RPL11,RPL10,RPL30,RPS2,RPS6KB2

Oxidative phosphorylation:
ATP5A1,ATP5D,ATP5G2,ATP5G1,ATP5G3,ATP5I,NDUFA11,NDUFS7,NDUFA2,ND6,NDUFA8,NDUFS2,NDUFS8,NDUFB2, NDUFV2,NDUFV3

Metabolism of carbohydrates:
SLC25A1,PCK1,SLC25A10,GALK1,GALT,PGLS,SLC37A4,AKR1B1,AKR1A1

Glucose metabolism:
SLC25A1,PCK1,SLC25A10,SLC37A4

Fatty acyl-CoA and cholesterol biosynthesis:
SLC25A1,PPT2,SLC27A3;
FDPS,MVD,DHCR7,PMVK,FDFT1,MVK

Globo Sphingolipid Metabolism:
ST3GAL1,ST6GALNAC4,
ST6GALNAC6,ST6GAL1

Biogenic Amine Synthesis:
DDC,ACHE,COMT

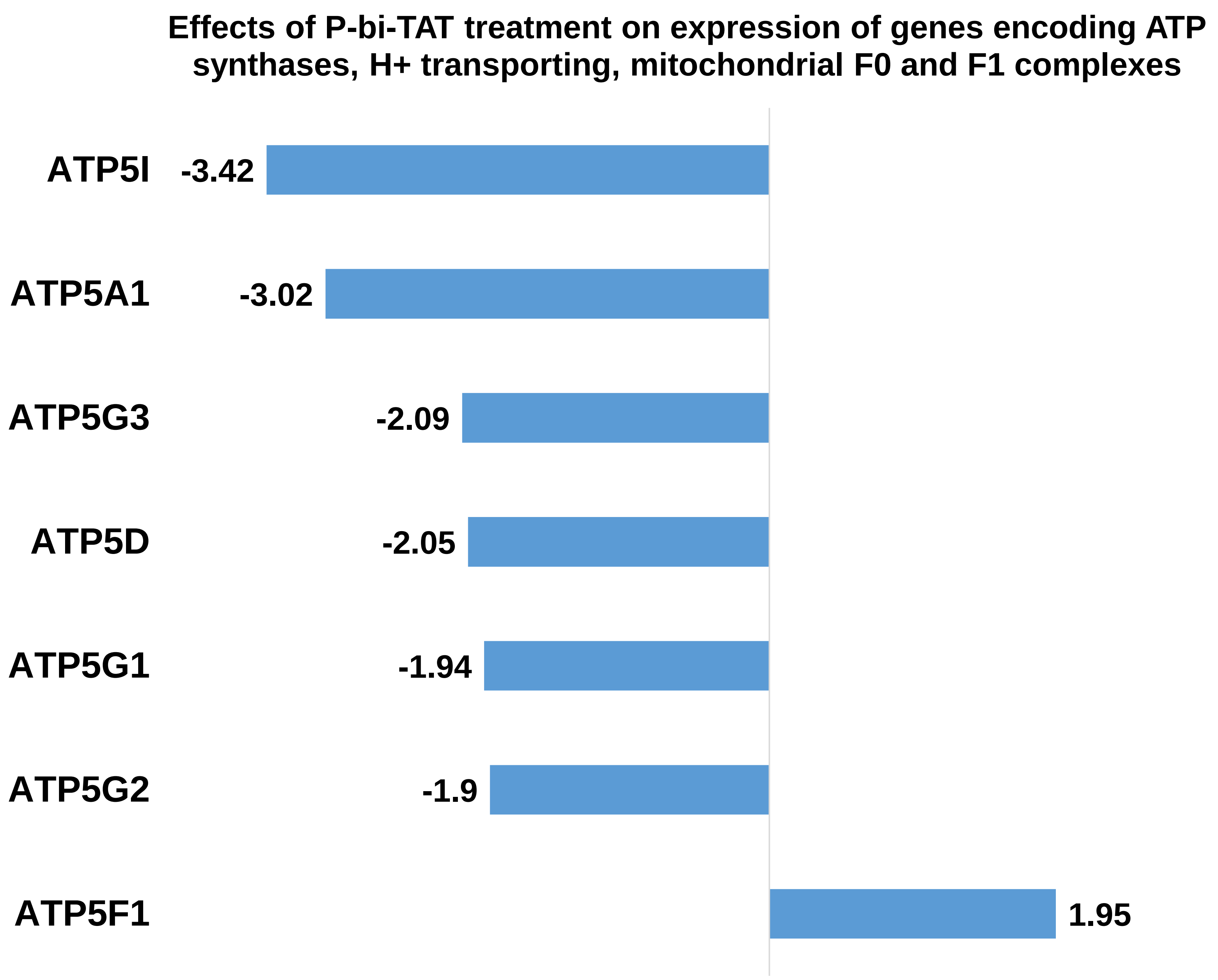
Effects of P-bi-TAT treatment on expression of genes encoding ATP synthases, H+ transporting, mitochondrial F0 and F1 complexes
ATP5I
-3.42
ATP5A1
-3.02
ATP5G3
-2.09
ATP5D
-2.05
ATP5G1
-1.94
ATP5G2
-1.9
ATP5F1
1.95

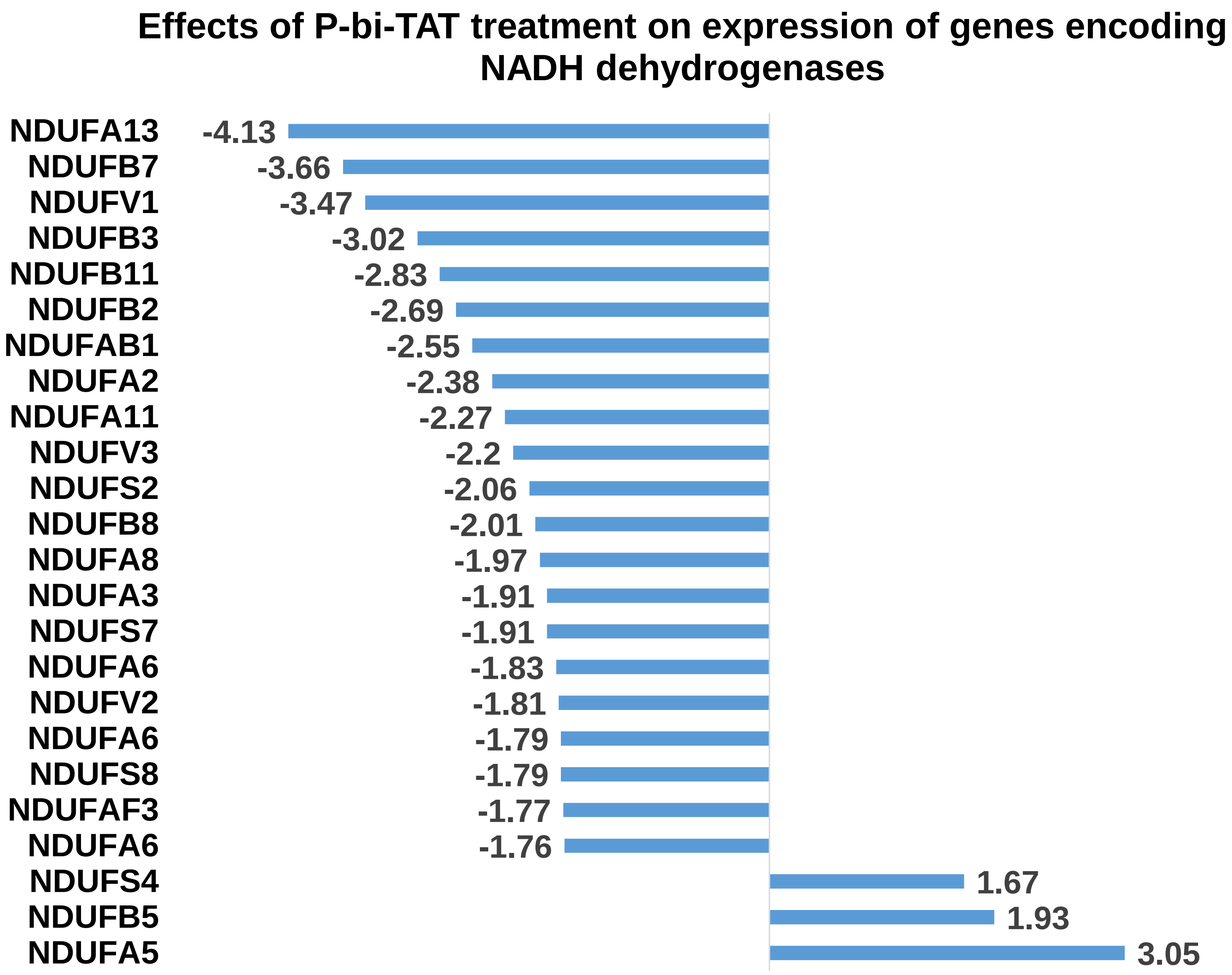
Effects of P-bi-TAT treatment on expression of genes encoding NADH dehydrogenases
NDUFA13 -4.13
NDUFB7 -3.66
NDUFV1 -3.47
NDUFB3 -3.02
NDUFB11 -2.83
NDUFB2 -2.69
NDUFAB1 -2.55
NDUFA2 -2.38
NDUFA11 -2.27
NDUFV3 -2.2
NDUFS2 -2.06
NDUFB8 -2.01
NDUFA8 -1.97
NDUFA3 -1.91
NDUFS7 -1.91
NDUFA6 -1.83
NDUFV2 -1.81
NDUFA6 -1.79
NDUFS8 -1.79
NDUFAF3 -1.77
NDUFA6 -1.76
NDUFS4 1.67
NDUFB5 1.93
NDUFA5 3.05

P-bi-TAT destroys the transcriptional architecture of the life-support infrastructure of cancer cells

P-bi-TAT targets the cellular membranes' structure and functions by inhibiting expression of G-protein-coupled receptors (GPCRs).

GENES SIGNIFICANTLY DOWN-REGULATED FOLLOWING THE P-bi-TAT TREATMENT

GPCR ligand binding:

XCR1,FPR1,RXFP3,PTGDR2,P2RY12,P2RY13,P2RY11,FFAR1,GHRHR,GPRC6A

GPCRs, Class A Rhodopsin-like:

DRD2,HRH3,BDKRB1,FPR1,XCR1,MC1R,NTSR1,OPRM1,SSTR5,OR1F1,OR1E1,OR1D2,OR5V1,OR2B3,
OR2J3,OR2H1,OR10H3,P2RY13,P2RY12,GPR4,P2RY11,CNR2,OR3A2,GPR171

GPCRs, Other:

TAAR2,GPR88,NTSR1,P2RY13,OR1F1,OR1E1,OR2H1,GHRHR,GRM1,P2RY11

Peptide GPCRs:

FPR1,BDKRB1,OPRM1,SSTR5,MC1R,NTSR1

GPCRs, Class C Metabotropic glutamate, pheromone:

GRM1,GPRC5C,GPRC5A

GPR40 Pathway:

PLCL1,PLCD3

G-protein-coupled receptors (GPCRs) are the largest and most diverse group of membrane receptors in eukaryotes. These cell surface receptors act like an inbox for messages in the form of light energy, peptides, lipids, sugars, and proteins. Such messages inform cells about the presence or absence of life-sustaining light or nutrients in their environment, or they convey information sent by other cells.
GPCRs play a key role in an incredible array of functions in the human body. Different GPCRs bind a tremendous variety of signaling molecules. Binding a signaling molecule to GPRCs causes a conformational change in the GPCR. This change then triggers the interaction between the GPCR and a nearby G protein.

P-bi-TAT destroys the transcriptional architecture of the life-support infrastructure of cancer cells

Interference with essential oncogenic pathways

GENES SIGNIFICANTLY DOWN-REGULATED FOLLOWING THE P-bi-TAT TREATMENT

Target Of Rapamycin (TOR) Signaling:
MLST8,CDC42,RHEB,ULK3,AKT1,PRR5L,EIF4EBP1

PIP3 activates AKT signaling:
BAD,RPS6KB2,AKT1

ErbB Signaling Pathway:
ERBB2,ARAF,BAD,SRC,JUN,HBEGF,HRAS,CBL,PAK4

RAF/MAP kinase cascade:
PHB,PEBP1,PPP5C

Hedgehog Signaling Pathway:
ADRBK1,ARRB2,KIF7

Telomere Maintenance:
NHP2,RUVBL2,FEN1

P-bi-TAT destroys the transcriptional architecture of the life-support infrastructure of cancer cells

Interference with chromatin remodeling and maintenance, transcription, and activated in cancer cells early-stage embryogenesis pathways

GENES SIGNIFICANTLY DOWN-REGULATED FOLLOWING THE P-bi-TAT TREATMENT

Histone Modifications:
H3F3B,HIST1H3D,HIST1H3J,HIST2H3A,HIST2H3C,HIST2H3D,DOT1L,EHMT2,SETDB1,SMYD5,HIST1H4C,HIST1H4H, HIST1H4I,HIST1H4J,HIST4H4,HIST1H3C,HIST1H3G,HIST1H4K,HIST1H4E,HIST1H3H,HIST1H4B,HIST1H4L,HIST1H3A, HIST1H4G,HIST1H3F,HIST1H3B,HIST1H4F,HIST1H3I,HIST1H4D,HIST1H4A,HIST1H3E

RNA Polymerase I Transcription:
TAF1C,POLR1D,POLR1C,PTRF,POLR2F,POLR2E,ZNRD1,POLR2L,POLR1E

Preimplantation Embryo Pathways:
BARX2,FOXD1,TPRX1,DDIT3,BATF3,TEAD4,HMGA1,SOX2

Olfactory receptor activity:
OR1E1,OR1F1,OR2H1,OR5V1,OR6C70,OR8K3,OR9G1,OR1L6,OR2T34,OR4K15,OR5P2,OR2B3,OR2G3,OR10H3, OR8B12,OR2J3,OR51I1,OR4D1,OR4M1,OR8H3,OR51E2,OR56A1,OR8U1,OR5L1,OR1D2,OR56B4,OR10A7,OR3A2, OR6C4,OR10J3,OR2AE1,OR2B11,OR51M1,OR2K2,OR5AP2,OR13G1,OR52M1,OR4S2,OR5AK2,OR8B3,OR5B2,OR1S2

GPCRs, Class A Rhodopsin-like:
DRD2,HRH3,BDKRB1,FPR1,XCR1,MC1R,NTSR1,OPRM1,SSTR5,OR1F1,OR1E1,OR1D2,OR5V1,OR2B3,OR2J3,OR2H1, OR10H3,P2RY13,P2RY12,GPR4,P2RY11,CNR2,OR3A2,GPR171

P-bi-TAT destroys the transcriptional architecture of the life-support infrastructure of cancer cells

Interference with networks implicated in collagen degradation, destruction of the extracellular matrix, and activation of metalloproteinases

GENES SIGNIFICANTLY DOWN-REGULATED FOLLOWING THE P-bi-TAT TREATMENT

Degradation of the extracellular matrix:
MMP19,BSG,CAST

Activation of Matrix Metalloproteinases:
TIMP1,MMP7,FURIN

Collagen degradation:
FURIN,MMP19

Matrix Metalloproteinases:
MMP7,MMP19,BSG,TIMP1

Biotransformation is the chemical modification (or modifications) made by an organism on a chemical compound.

Biotransformation means chemical alteration of chemicals such as nutrients, amino acids, toxins, and drugs in the body.

Phase I reactions convert a parent drug to more polar (water soluble) **active metabolites** by unmasking or inserting a polar functional group (-OH, -SH, -NH2)

Phase II reactions convert a parent drug to more polar (water soluble) **inactive metabolites** by conjugation of subgroups to -OH, -SH, -NH2 functional groups on drug

Expression of genes regulating the BIOTRANSFRMATION METAPATHWAY is markedly affected by the P-bi-TAT treatment

UP-regulated (10 genes):
CYP1B1,FMO4,CYP4V2,CYP51A1,KCNAB3,GSTCD,MGST1,HS2ST1,HS3ST3A1,HS6ST3

Down-regulated (25 genes):
CYP3A5,AKR1A1,AKR7A3,EPHX1,COMT,NNMT, CYP19A1,AKR1D1,AKR1B1,KCNAB1,GPX1,GPX4, GSTM2,GSTM3,GSTM4,GSTO1,GSTT2,GSTT2B, GSTZ1,MGST3,GSS,NAT14,CHST7,CHST10,NDST1

Phase I reactions convert a parent drug to more polar (water soluble) **active metabolites** by unmasking or inserting a polar functional group (-OH, -SH, -NH2)

Phase I biotransformation genes are predominantly **UP-REGULATED** by P-bi-TAT: CYP51A1,CYP1B1,CYP4V2; CES1,ESD,LIPA,PON2

CONCLUSION: P-bi-TAT would facilitate **MORE EFFICIENT** drug conversion to **ACTIVE METABOLITES**

Phase II reactions convert a parent drug to more polar (water soluble) **inactive metabolites** by conjugation of subgroups to -OH, -SH, -NH2 functional groups on drug

Phase II biotransformation genes are predominantly **DOWN-REGULATED** by P-bi-TAT: COMT,NNMT,PNP,AKR1A1

CONCLUSION: P-bi-TAT would facilitate **LESS EFFICIENT** drug conversion to **INACTIVE METABOLITES**

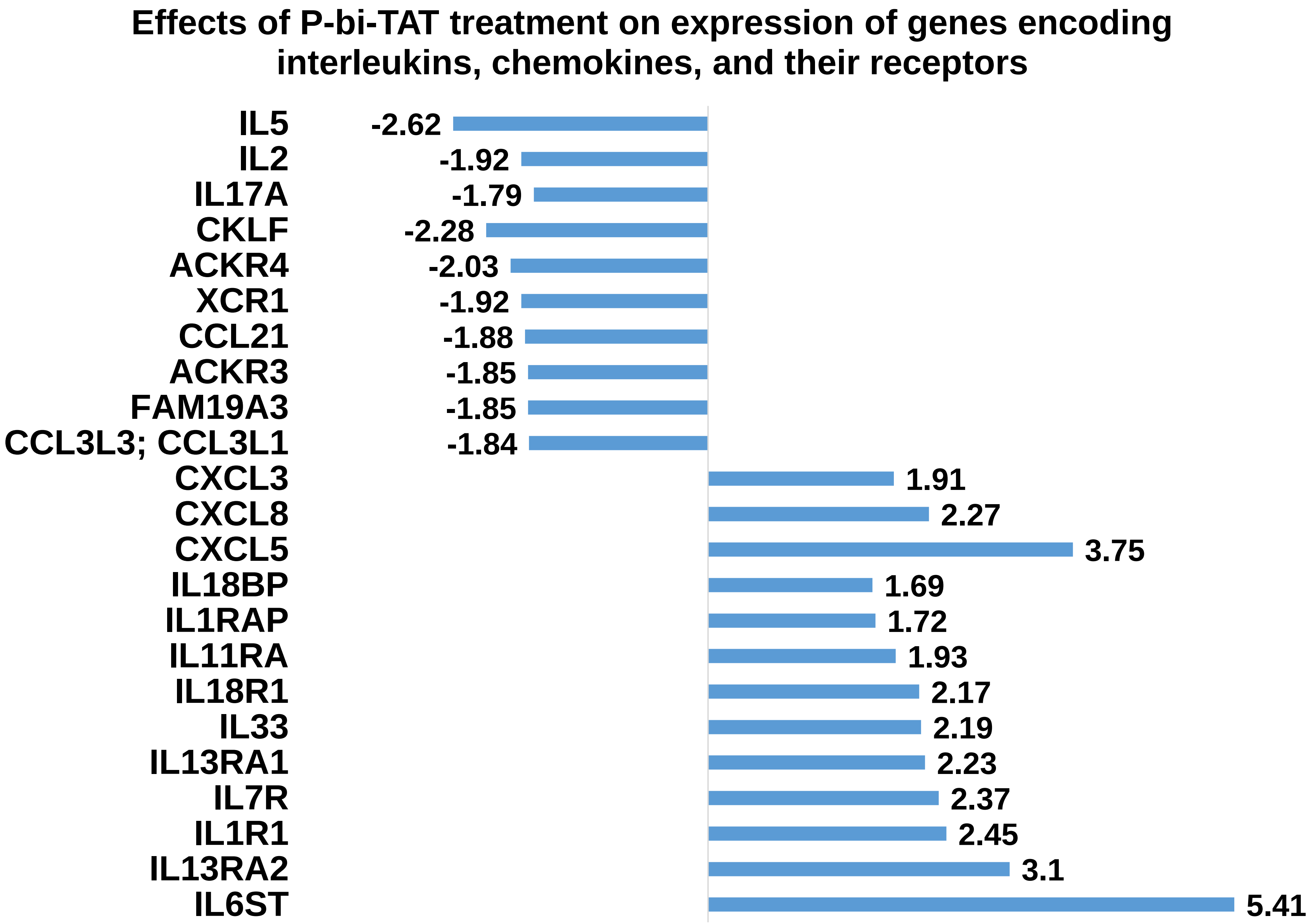
Effects of P-bi-TAT treatment on expression of genes encoding interleukins, chemokines, and their receptors
IL5
-2.62
IL2
-1.92
IL17A
-1.79
CKLF
-2.28
ACKR4
-2.03
XCR1
-1.92
CCL21
-1.88
ACKR3
-1.85
FAM19A3
-1.85
CCL3L3; CCL3L1
-1.84
CXCL3
1.91
CXCL8
2.27
CXCL5
3.75
IL18BP
1.69
IL1RAP
1.72
IL11RA
1.93
IL18R1
2.17
IL33
2.19
IL13RA1
2.23
IL7R
2.37
IL1R1
2.45
IL13RA2
3.1
IL6ST
5.41

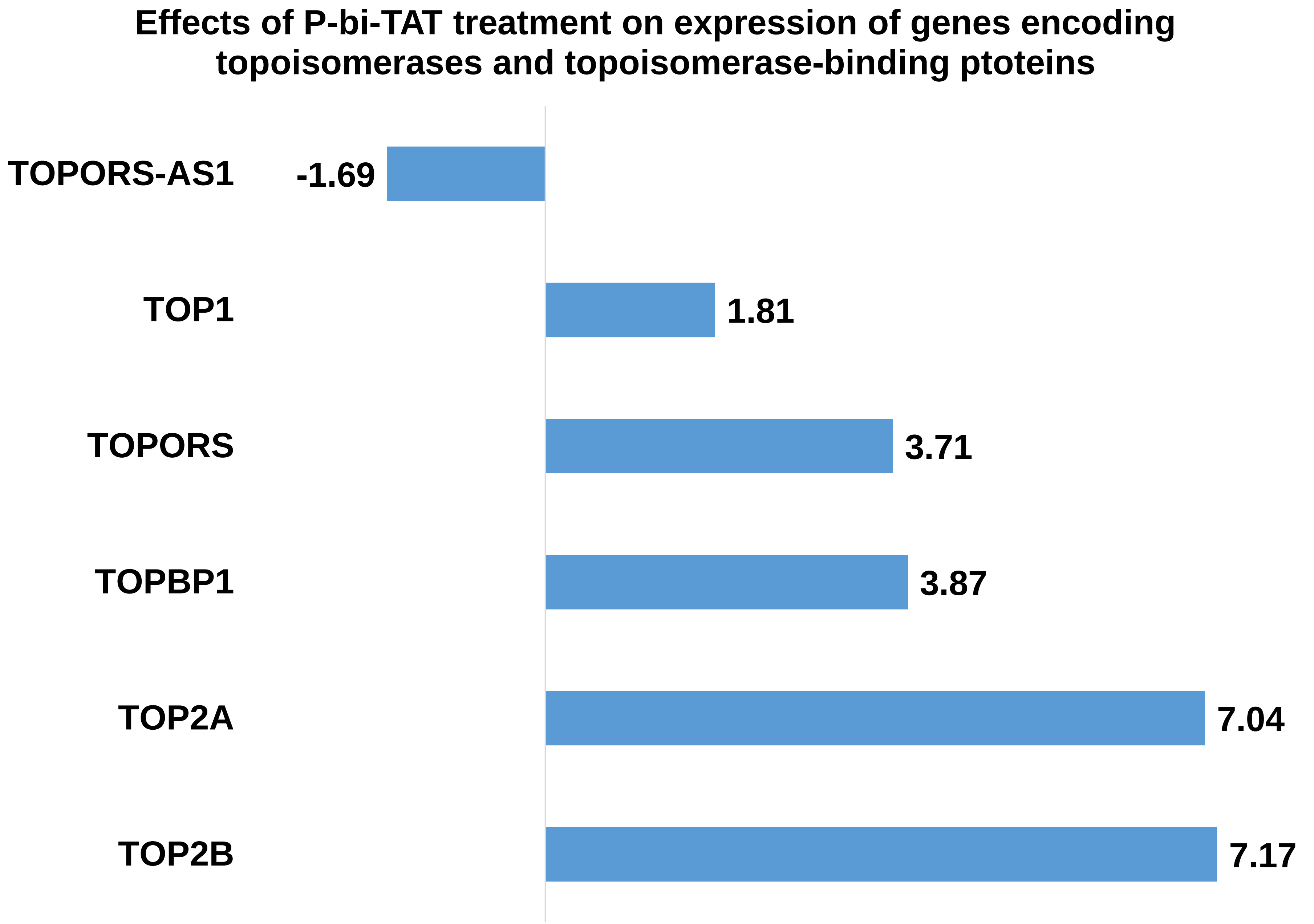
Effects of P-bi-TAT treatment on expression of genes encoding topoisomerases and topoisomerase-binding ptoteins
TOPORS-AS1
-1.69
TOP1
1.81
TOPORS
3.71
TOPBP1
3.87
TOP2A
7.04
TOP2B
7.17
