# Supplementary material for: Effects of Anticancer Agent P-bi-TAT on Gene Expression Link the Integrin Thyroid Hormone Receptor to Expression of Stemness and Energy Metabolism Genes in Cancer Cells
Source: Metabolites. 2022 Apr 4;12(4):325. doi: 10.3390/metabo12040325 (PMC9029602; doi:10.3390/metabo12040325)
Supplement: Supplementary file 1 [file metabolites-12-00325-s001.zip › Supp Summary S2. P_bi_TAT effects on gene expression.pdf]

STUDY OVERVIEW

Human cancer cell line models:

**Primary human glioblastoma cells**	**GBM 021913**
**Pancreatic cancer cell line**	**SUIT2-luc**

- **Treated with 30 μM of P-bi-TAT (n = 3)**
- **After 24 hours, cells were collected in tri reagent**
- **Samples given to Microarray core facility for gene expression study (Using Affymetrix Protocol for Clariom S Microarrays)**
- **No measurable effects on cells' morphology, growth, and viability were observed after 24 hours treatment with 30 μM of P-bi-TAT**

Filter criteria:

**Fold change**	**>1.5 or < -1.5**
**P - Value**	**< 0.05**

STUDY SUMMARY

Effect of the P-bi-TAT treatment on gene expression

Cell line	Number of significantly affected genes	Up-regulated	Down-regulated	Number of significantly affected pathways	Number of affected genes in pathways
SUIT2-luc	1348	825	523	39	4 - 29
GBM 021913	5689	3277	2412	250	4 - 180

Filter criteria:

Fold change	>1.5 or < -1.5
P - Value	< 0.05

Two significantly affected cell lines were selected for identification of the consensus gene expression signature and commonly affected signaling pathways

- **Primary human glioblastoma cells** GBM 021913
- **Human metastatic pancreatic cancer cell line** SUIT2-luc

Effect of the P-bi-TAT treatment on gene expression

Cell line	Number of significantly affected genes	Up-regulated	Down-regulated	Number of significantly affected pathways	Number of affected genes in pathways
SUIT2-luc	1348	825	523	39	4 - 29
GBM 021913	5689	3277	2412	250	4 - 180

CONSENSUS SET OF 737 GENES AFFECTED BY P-bi-TAT THERAPY

Primary human glioblastoma cells GBM 021913

Human metastatic pancreatic cancer cell line SUIT2-luc

CONSENSUS SET OF 737 GENES AFFECTED BY P-bi-TAT THERAPY

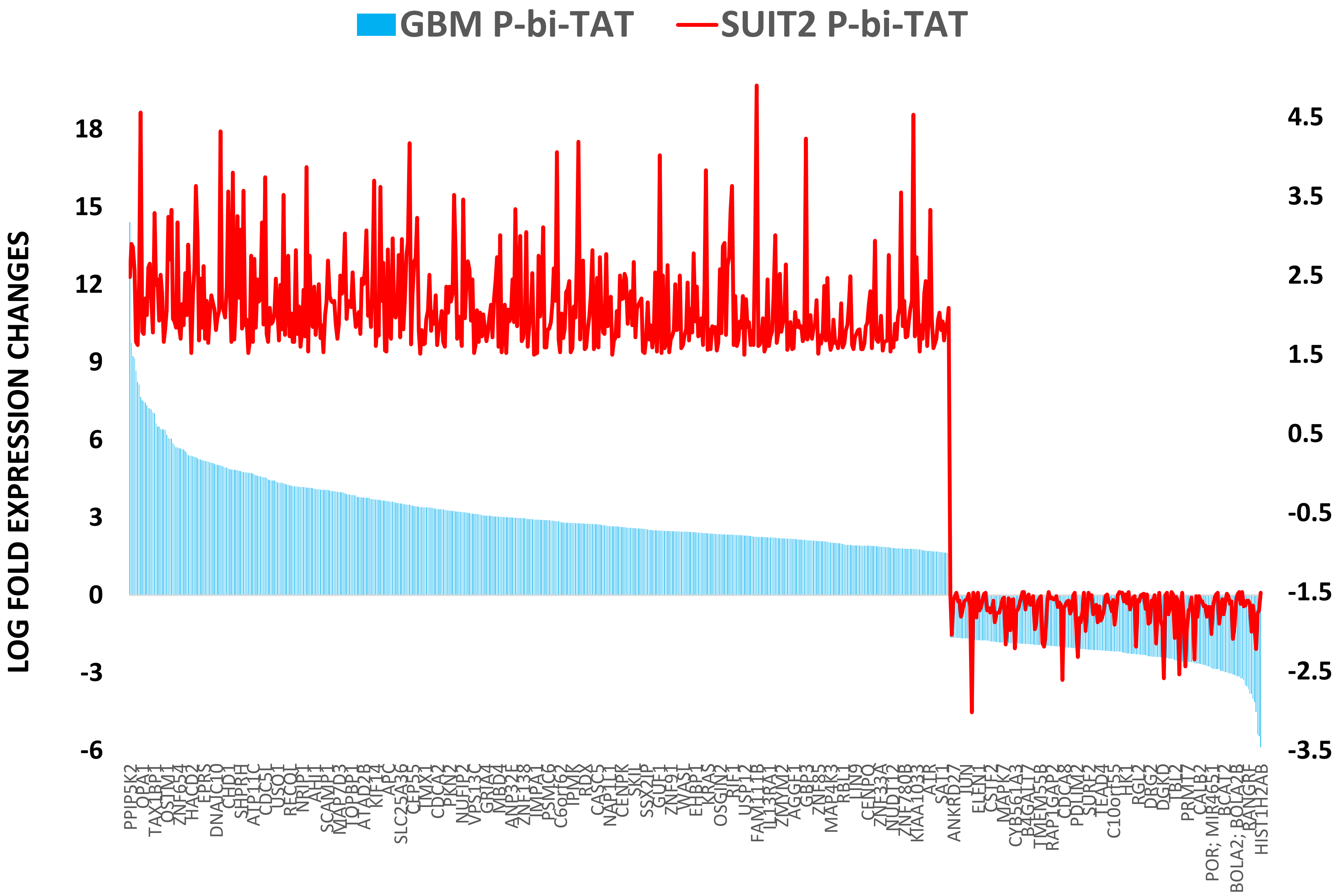

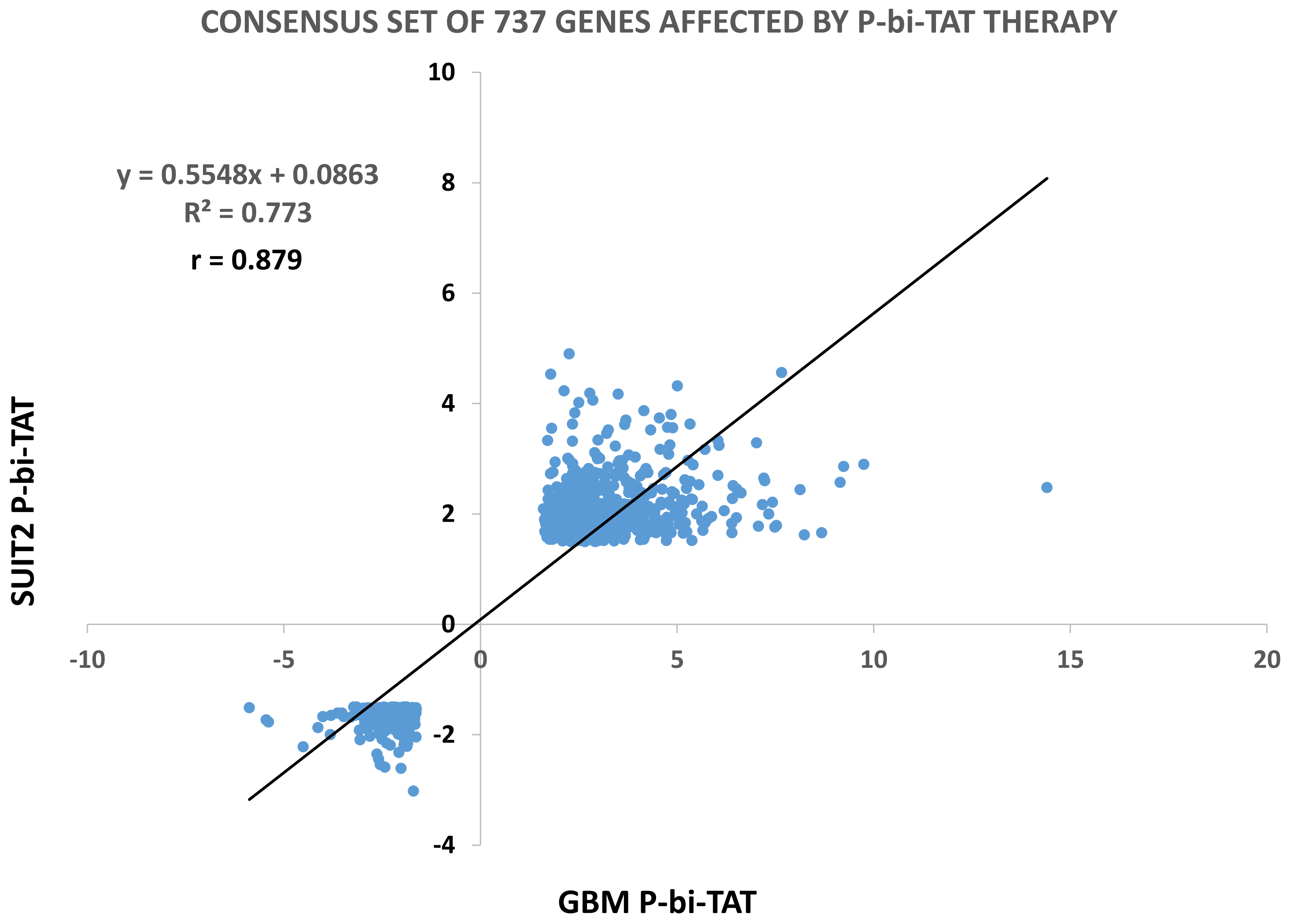

CONSENSUS SET OF 737 GENES AFFECTED BY P-bi-TAT THERAPY
y = 0.5548x + 0.0863
R² = 0.773
r = 0.879
10
8
6
4
2
0
-2
-4
-10
-5
0
5
10
15
20
SUIT2 P-bi-TAT
GBM P-bi-TAT

Effect of the P-bi-TAT treatment on gene expression

Cell line	Number of significantly affected genes	Up-regulated	Down-regulated	Number of significantly affected pathways	Number of affected genes in pathways
SUIT2-luc	1348	825	523	39	4 - 29
GBM 021913	5689	3277	2412	250	4 - 180

COMMON SIGNALING PATHWAYS

Effect of the P-bi-TAT treatment on gene expression
SIXTEEN COMMON PATHWAYS

Pathway	Cell line	Up-regulated	Down-regulated	Total genes	p-value	Cell line	Up-regulated	Down-regulated	Total genes	p-value
VEGFA-VEGFR2 Signaling Pathway	GBM	51	-34	85	0.011056	SUIT2-luc	14	-15	29	0.00245
Androgen receptor signaling pathway	GBM	28	-15	43	0.000046	SUIT2-luc	9	-4	13	0.00843
Brain-Derived Neurotrophic Factor (BDNF) signaling pathway	GBM	36	-18	54	0.020347	SUIT2-luc	9	-10	19	0.00678
Deubiquitination	GBM	12	-1	13	0	SUIT2-luc	4	0	4	0
Endoderm Differentiation	GBM	41	-12	53	0.025638	SUIT2-luc	11	-6	17	0.02808
Focal Adhesion	GBM	37	-32	69	0.011855	SUIT2-luc	15	-9	24	0.00308
Gastric Cancer Network 2	GBM	11	-4	15	0.027934	SUIT2-luc	7	0	7	0.00467
Human Thyroid Stimulating Hormone (TSH) signaling pathway	GBM	17	-13	30	0.002383	SUIT2-luc	6	-4	10	0.01235
IL-6 signaling pathway	GBM	16	-6	22	0.001737	SUIT2-luc	7	-2	9	0.00193
Integrin-mediated Cell Adhesion	GBM	20	-20	40	0.013987	SUIT2-luc	9	-5	14	0.0085
Interleukin-11 Signaling Pathway	GBM	17	-10	27	0.000004	SUIT2-luc	4	-4	8	0.00831
MAPK Signaling Pathway	GBM	8	-8	16	0.002772	SUIT2-luc	13	-8	21	0.00768
Olfactory receptor activity	GBM	5	-42	47	0	SUIT2-luc	1	-5	6	4E-06
Signaling of Hepatocyte Growth Factor Receptor	GBM	11	-5	16	0.020177	SUIT2-luc	3	-3	6	0.02432
TCF dependent signaling in response to WNT	GBM	11	-3	14	0	SUIT2-luc	4	-5	9	0.00539
TGF-beta Signaling Pathway	GBM	48	-13	61	0.000019	SUIT2-luc	12	-5	17	0.01388

VEGFA-VEGFR2 Signaling Pathway is the most significantly affected pathway in U87 human glioblastoma cell line as well, suggesting that antiangiogenic effect may represent one of the main biological effects of the P-bi-TAT therapy

Effect of the P-bi-TAT treatment on gene expression

Cell line	Number of significantly affected genes	Up-regulated	Down-regulated	Number of significantly affected pathways	Number of affected genes in pathways
SUIT2-luc	1348	825	523	39	4 - 29
GBM 021913	5689	3277	2412	250	4 - 180

SIGNALING "CHAOS" IN CANCER CELLS

MOLECULAR INTERFERENCE MODEL OF THE P-bi-TAT THERAPEUTIC EFFECTS ON HUMAN CANCER CELLS

P-bi-TAT TREATMENT OF HUMAN CANCER CELLS CAUSED MARKED DISRUPTIONS OF EXPRESSION OF THOUSANDS GENES IMPLICATED IN FUNCTIONS OF HUNDREDS SIGNALING PATHWAYS. WITHIN MOST AFFECTED PATHWAYS, THE SIGNIFICANT GENE EXPRESSION CHANGES WERE DOCUMENTED FOR BOTH UP-REGULATED AND DOWN-REGULATED TRANSCRIPTS FOLLOWING THE P-bi-TAT ADMINISTRATION. THESE DATA SUGGEST THAT THE P-bi-TAT TREATMENT CAUSES THE MOLECULAR INTERFERENCE WITH MULTIPLE SIGNILING PATHWAYS, WHICH RESULTS IN AN APARENT SIGNALING “CHAOS” IN CANCER CELLS.

In contrast to most affected signaling pathways, deubiquitination pathway appears predominantly activated, thus representing one notable exception from this rule. However, activation of the deubiquitination pathway would interfere with normal turnover of proteins, including pathway’s receptors and down-stream signal-transducing molecules, which is required for the proper sustained functions of signaling pathways. Therefore, activation of deubiquitination pathway following the P-bi-TAT treatment would be consistent with the molecular interference model.

SIGNALING "CHAOS" IN GLIOBLASTOMA MULTIFORME CELLS

46 pathways (number of affected genes from 21 to 180)

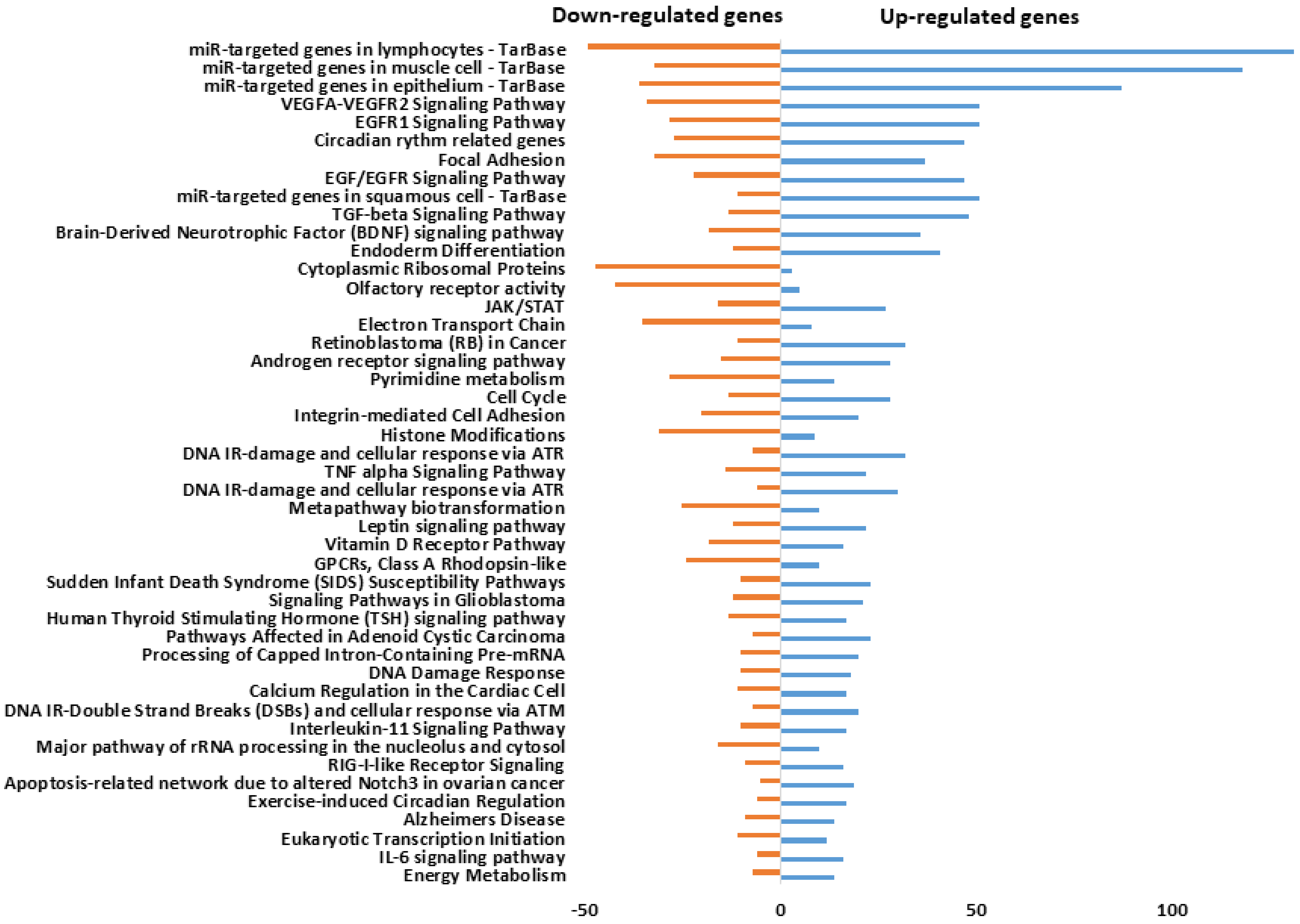

SIGNALING "CHAOS" IN GLIOBLASTOMA MULTIFORME CELLS

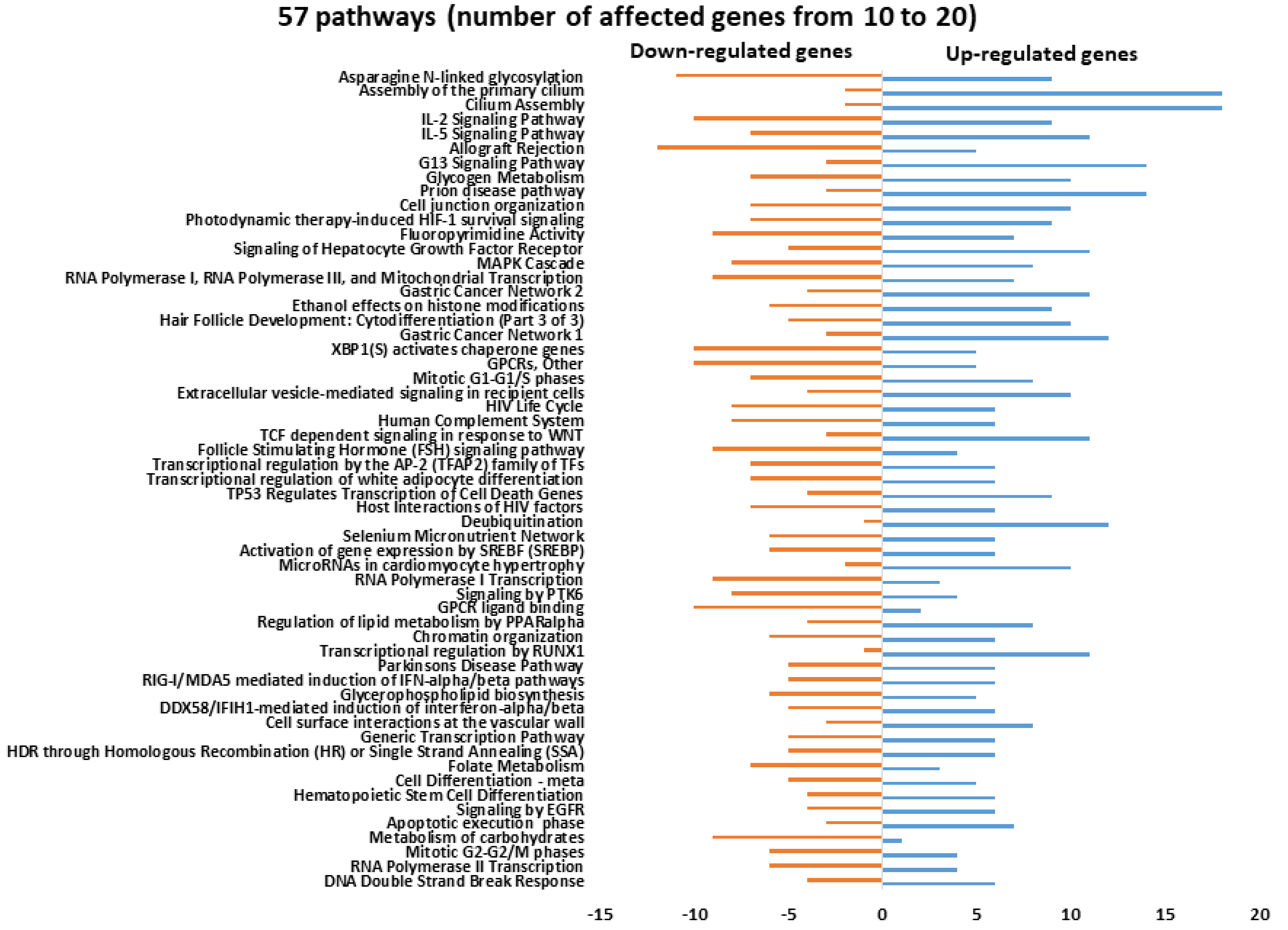

INTEGRIN CROSSLINKING MODEL OF THE P-bi-TAT THERAPETIC EFFECTS ON HUMAN CANCER CELLS

Integrin's interactions with extracellular matrix proteins facilitate fine 3D placements of integrin molecules embedded within the lipid bilayer of cellular membranes, which is required for integrin's activation and signaling cross-talks with a multitude of specific signaling pathways. We propose that unique features of the molecular structure of the P-bi-TAT allow for the efficient interference with and disruption of these processes by causing, in effect, the “crosslinking” of integrin molecules. It alters dynamics of integrin’s mobility, turnover, and proper 3D placements within the cellular membrane causing the signaling “chaos” in target cells.

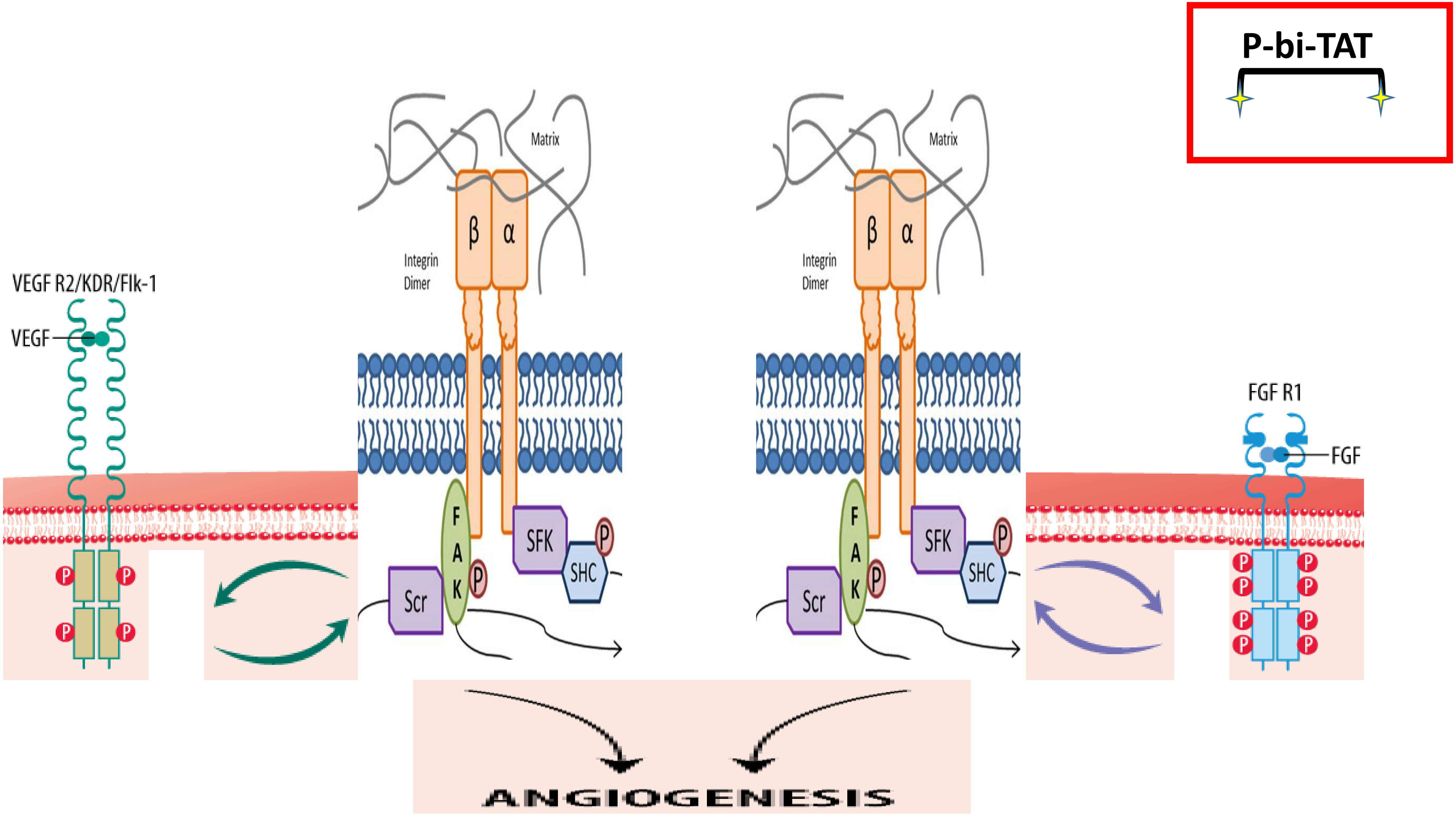
P-bi-TAT
VEGF R2/KDR/Flk-1
VEGF
Matrix
Integrin
Dimer
β
α
F
A
K
P
SFK
SHC
Scr
FGF R1
FGF
ANGIOGENESIS

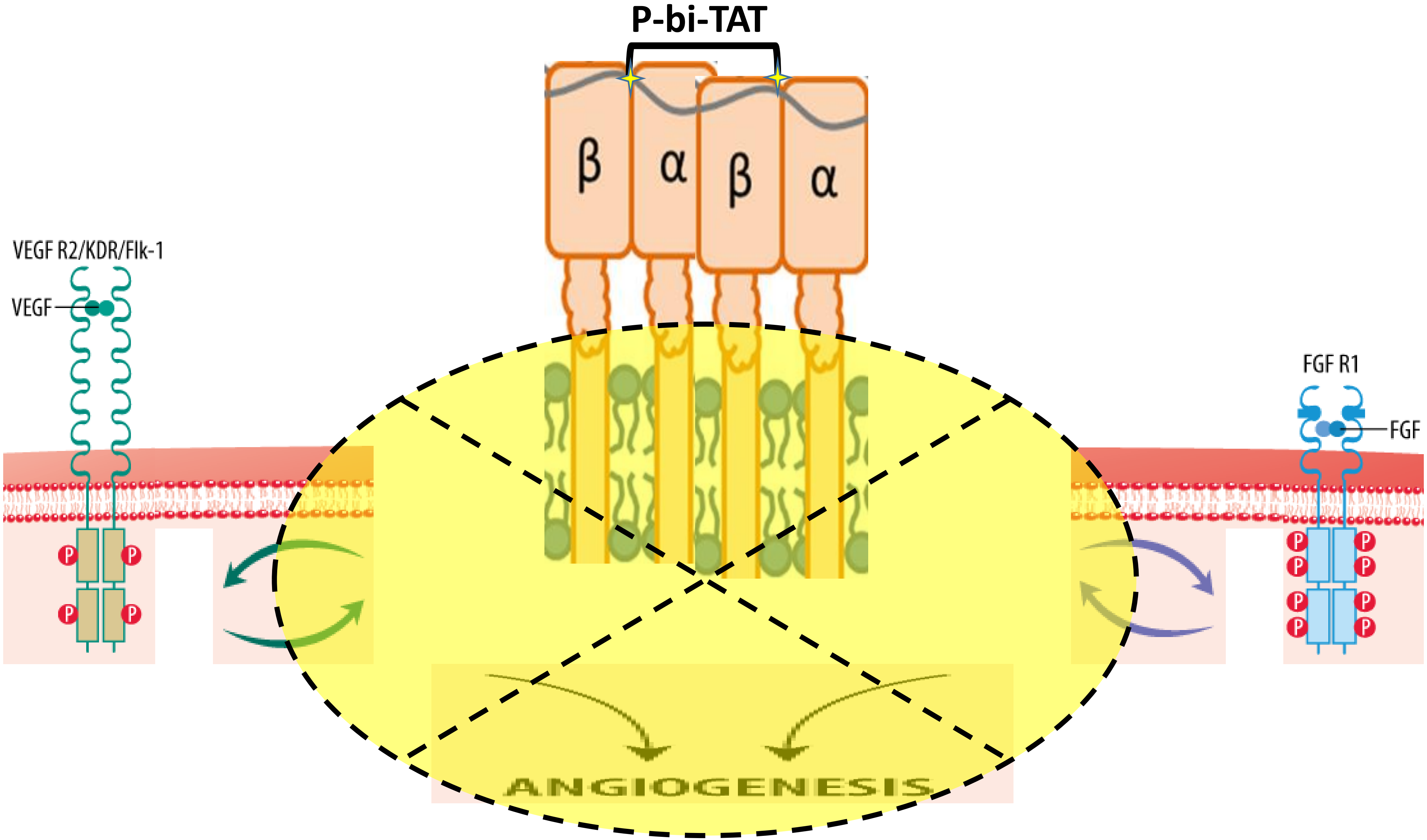

P-bi-TAT
β
α
β
α
VEGF R2/KDR/Flk-1
VEGF
FGF R1
FGF
ANGIOGENESIS

P-bi-TAT nano-therapeutics

Effect on gene expression in primary human Glioblastoma multiforme (GBM) cells GBM 021913 established as the primary cell line from the patient’s tumor

103 pathways significantly affected by treatment with P-bi-TAT for 24 hrs

($p < 0.05$; number of affected genes from 10 to 180)

250 pathways significantly affected by treatment with P-bi-TAT for 24 hrs

($p < 0.05$; number of affected genes from 4 to 180)

Effect on gene expression in primary human Glioblastoma multiforme (GBM) cells GBM 021913

treated vs untreated

- treated: 2 samples, untreated: 2 samples

Filter criteria:

- Fold Change: > 1.5 or < -1.5
- P-val: < 0.05

Total number of genes: 21448

- Genes passed filter criteria: 5689 (26.52%)
  - Up-Regulated: 3277 (57.6%)
  - Down-Regulated: 2412 (42.4%)

Up-Regulated (3277)

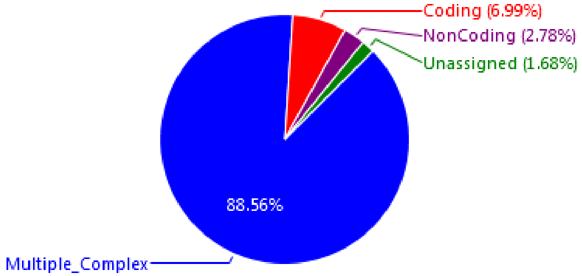

Down-Regulated (2412)

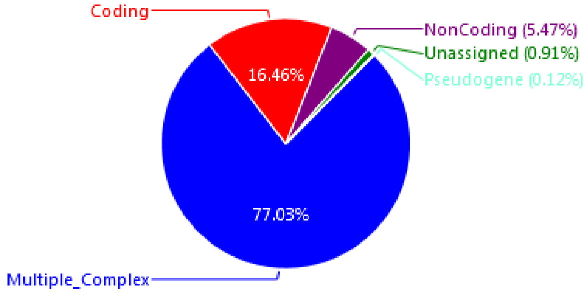

Group	Total	Passed Filter	Up-Regulated	Down-Regulated
Multiple_Complex	16081	4760	2902	1858
Coding	3878	626	229	397
NonCoding	1116	223	91	132
Unassigned	352	77	55	22
Pseudogene	21	3	0	3

GENE GROUP DEFINITIONS

Group	Definition
Multiple_ complex	Gene contains more than one locus
Coding	Gene contains only protein-coding transcripts
Noncoding	Gene contains only non-protein-coding transcripts. Most of these are long non-coding genes, but occasionally they may be small non-coding genes where the genetic constitution cannot be determined.
Unassigned	Locus type could not be determined based on source data
Pseudogene	Gene contains only represents pseudogenes (imperfect copy of a gene).

Multiple_Complex (16081)

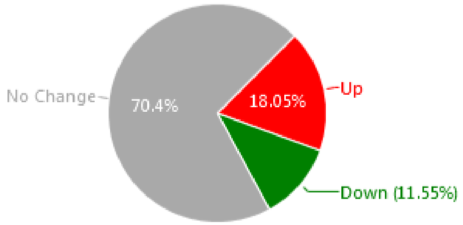

Coding (3878)

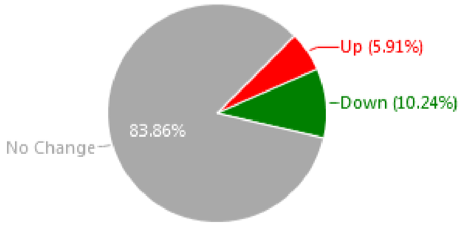

NonCoding (1116)

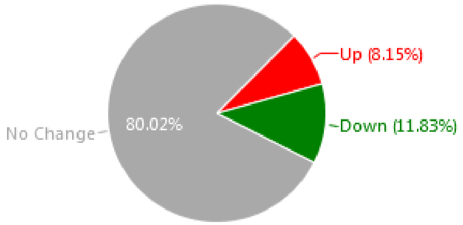

Unassigned (352)

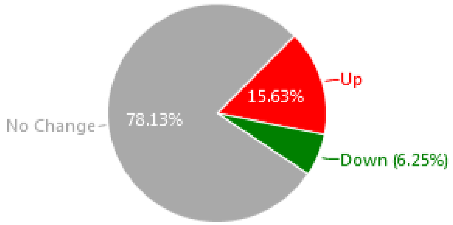

Pseudogene (21)

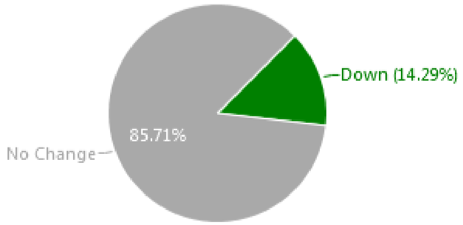

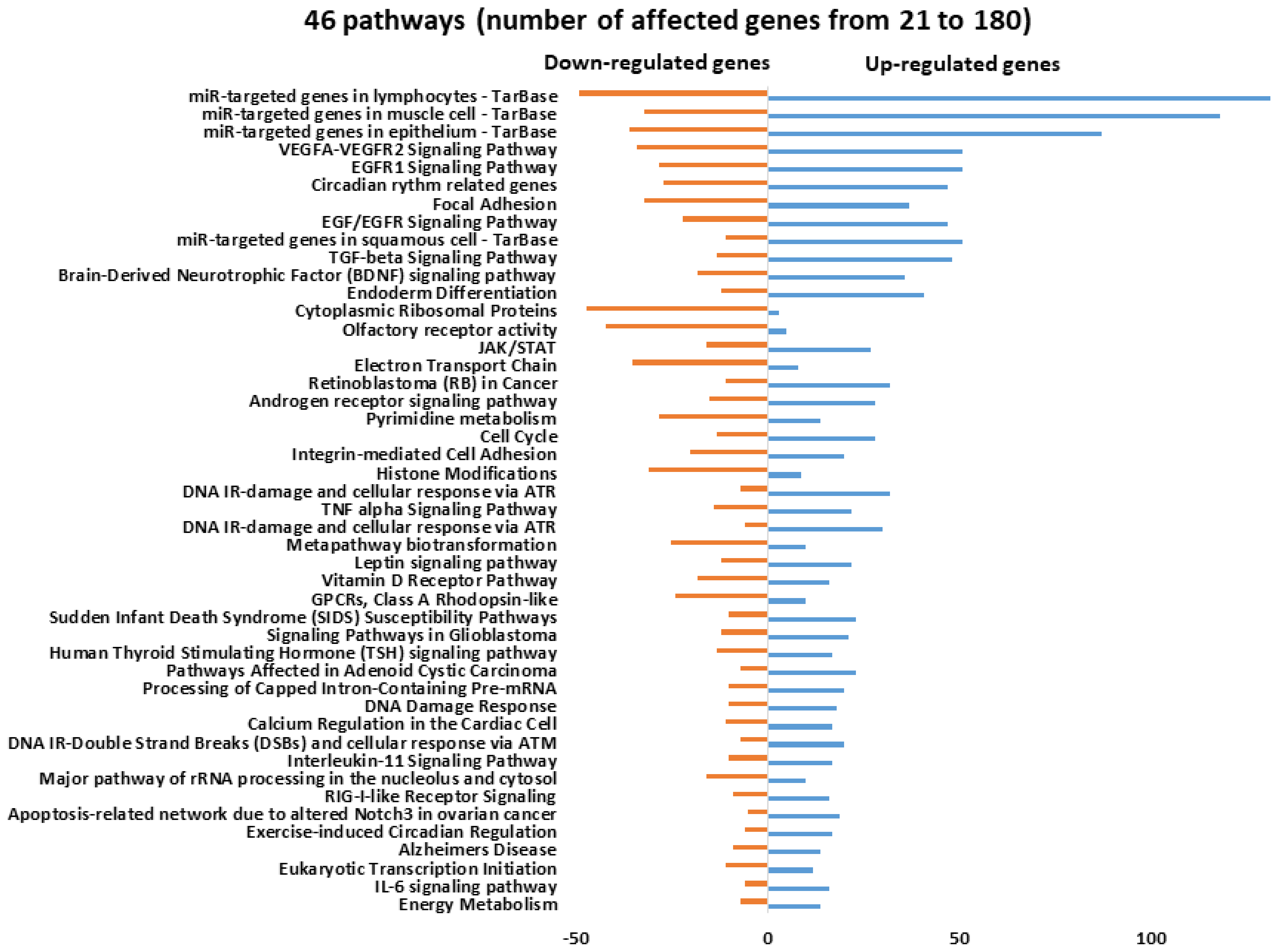
46 pathways (number of affected genes from 21 to 180)
Down-regulated genes
Up-regulated genes
miR-targeted genes in lymphocytes - TarBase
miR-targeted genes in muscle cell - TarBase
miR-targeted genes in epithelium - TarBase
VEGFA-VEGFR2 Signaling Pathway
EGFR1 Signaling Pathway
Circadian rythm related genes
Focal Adhesion
EGF/EGFR Signaling Pathway
miR-targeted genes in squamous cell - TarBase
TGF-beta Signaling Pathway
Brain-Derived Neurotrophic Factor (BDNF) signaling pathway
Endoderm Differentiation
Cytoplasmic Ribosomal Proteins
Olfactory receptor activity
JAK/STAT
Electron Transport Chain
Retinoblastoma (RB) in Cancer
Androgen receptor signaling pathway
Pyrimidine metabolism
Cell Cycle
Integrin-mediated Cell Adhesion
Histone Modifications
DNA IR-damage and cellular response via ATR
TNF alpha Signaling Pathway
DNA IR-damage and cellular response via ATR
Metapathway biotransformation
Leptin signaling pathway
Vitamin D Receptor Pathway
GPCRs, Class A Rhodopsin-like
Sudden Infant Death Syndrome (SIDS) Susceptibility Pathways
Signaling Pathways in Glioblastoma
Human Thyroid Stimulating Hormone (TSH) signaling pathway
Pathways Affected in Adenoid Cystic Carcinoma
Processing of Capped Intron-Containing Pre-mRNA
DNA Damage Response
Calcium Regulation in the Cardiac Cell
DNA IR-Double Strand Breaks (DSBs) and cellular response via ATM
Interleukin-11 Signaling Pathway
Major pathway of rRNA processing in the nucleolus and cytosol
RIG-I-like Receptor Signaling
Apoptosis-related network due to altered Notch3 in ovarian cancer
Exercise-induced Circadian Regulation
Alzheimers Disease
Eukaryotic Transcription Initiation
IL-6 signaling pathway
Energy Metabolism
-50
0
50
100

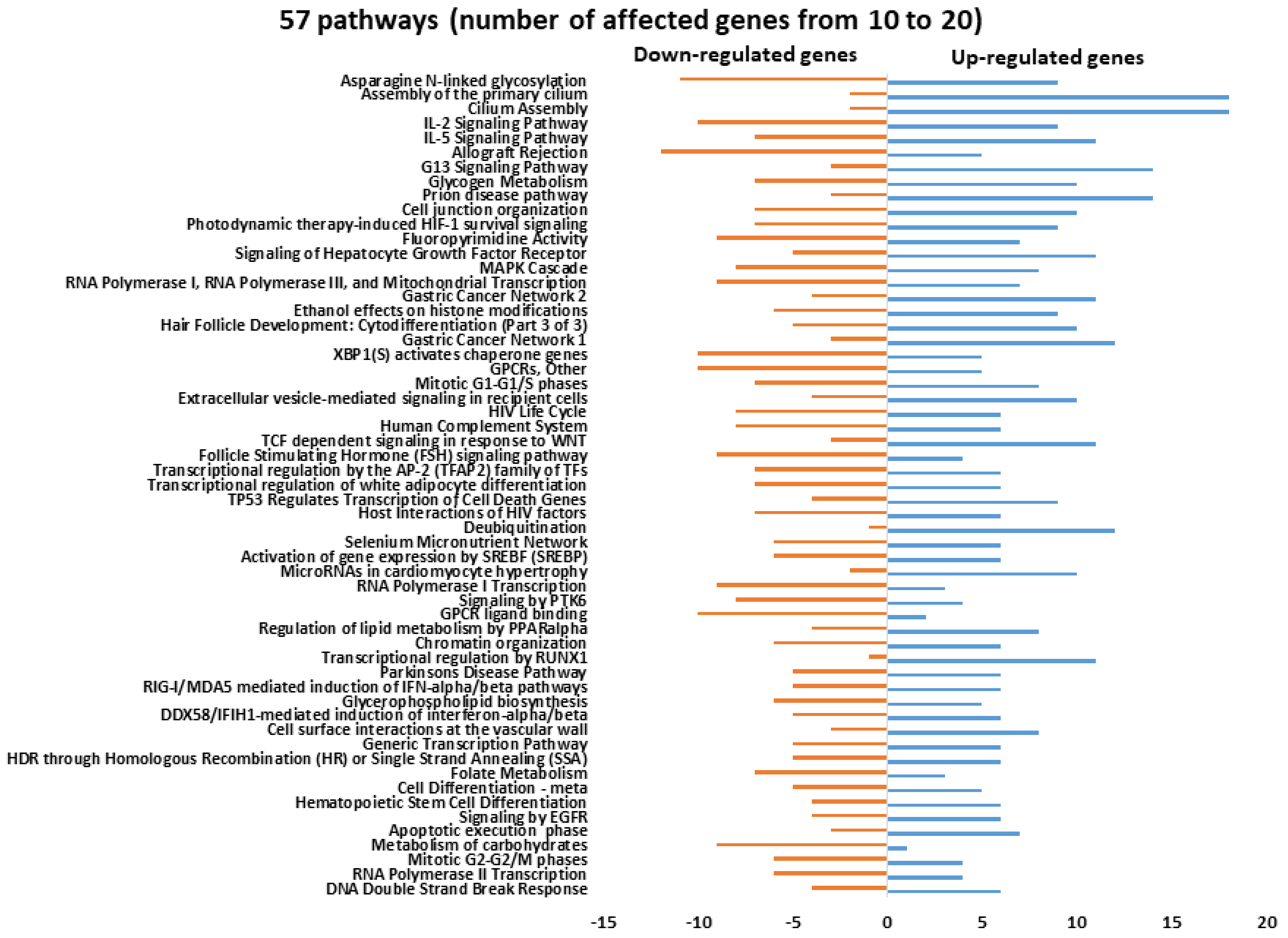
57 pathways (number of affected genes from 10 to 20)
Down-regulated genes
Up-regulated genes
Asparagine N-linked glycosylation
Assembly of the primary cilium
Cilium Assembly
IL-2 Signaling Pathway
IL-5 Signaling Pathway
Allograft Rejection
G13 Signaling Pathway
Glycogen Metabolism
Prion disease pathway
Cell junction organization
Photodynamic therapy-induced HIF-1 survival signaling
Fluoropyrimidine Activity
Signaling of Hepatocyte Growth Factor Receptor
MAPK Cascade
RNA Polymerase I, RNA Polymerase III, and Mitochondrial Transcription
Gastric Cancer Network 2
Ethanol effects on histone modifications
Hair Follicle Development: Cytodifferentiation (Part 3 of 3)
Gastric Cancer Network 1
XBP1(S) activates chaperone genes
GPCRs, Other
Mitotic G1-G1/S phases
Extracellular vesicle-mediated signaling in recipient cells
HIV Life Cycle
Human Complement System
TCF dependent signaling in response to WNT
Follicle Stimulating Hormone (FSH) signaling pathway
Transcriptional regulation by the AP-2 (TFAP2) family of TFs
Transcriptional regulation of white adipocyte differentiation
TP53 Regulates Transcription of Cell Death Genes
Host Interactions of HIV factors
Deubiquitination
Selenium Micronutrient Network
Activation of gene expression by SREBF (SREBP)
MicroRNAs in cardiomyocyte hypertrophy
RNA Polymerase I Transcription
Signaling by PTK6
GPCR ligand binding
Regulation of lipid metabolism by PPARalpha
Chromatin organization
Transcriptional regulation by RUNX1
Parkinsons Disease Pathway
RIG-I/MDA5 mediated induction of IFN-alpha/beta pathways
Glycerophospholipid biosynthesis
DDX58/IFIH1-mediated induction of interferon-alpha/beta
Cell surface interactions at the vascular wall
Generic Transcription Pathway
HDR through Homologous Recombination (HR) or Single Strand Annealing (SSA)
Folate Metabolism
Cell Differentiation - meta
Hematopoietic Stem Cell Differentiation
Signaling by EGFR
Apoptotic execution phase
Metabolism of carbohydrates
Mitotic G2-G2/M phases
RNA Polymerase II Transcription
DNA Double Strand Break Response
-15
-10
-5
0
5
10
15
20

Effect of the P-bi-TAT treatment on gene expression
FGF RECEPTORS' PATHWAYS

Pathway	Total genes	Up-regulated	Up List	Down-regulated	Down List	Significance	p-value
Signaling by FGFR2	8	6	FRS2,PTPN11,PIK3R1,PIK3CA,BRAF,HNRNPA1	2	CBL,PTBP1	15.96	0
Signaling by FGFR3	7	6	FRS2,PTPN11,BRAF,PIK3R1,PIK3CA,GALNT3	1	CBL	9.88	0
Signaling by FGFR1	6	5	PTPN11,FRS2,PIK3R1,PIK3CA,BRAF	1	CBL	12.34	0
Signaling by FGFR4	6	5	FRS2,BRAF,PTPN11,PIK3R1,PIK3CA	1	CBL	6.15	0.000001
