# Supplementary material for: Effects of Anticancer Agent P-bi-TAT on Gene Expression Link the Integrin Thyroid Hormone Receptor to Expression of Stemness and Energy Metabolism Genes in Cancer Cells
Source: Metabolites. 2022 Apr 4;12(4):325. doi: 10.3390/metabo12040325 (PMC9029602; doi:10.3390/metabo12040325)
Supplement: Supplementary file 1 [file metabolites-12-00325-s001.zip › Supp Summary S3. P_bi_TAT Cancer Survival Pathways.pdf]

EFFECT OF P-bi-TAT TREATMENT ON EXPRESSION OF HUMAN CANCER SURVIVAL GENES

Human cancer survival predictor genes are genes expression of which is highly significantly associated with long-term clinical outcomes in cancer patients such as 5-year survival and cancer-specific death. This highly relevant clinically category of genes was identified in a recent study of global gene expression patterns of all protein-coding genes in the clinical samples of 33 different human cancers from 9,666 patients of The Cancer Genome Atlas (TCGA) and the gene expression patterns in 37 normal human tissues obtained from 162 healthy subjects in the Human Protein Atlas (HPA) project (Uhlen et al., Science 357, 660; 2017; published on August 18, 2017). In total, more than 100 million Kaplan-Meier plots were generated that corresponded to all 19,571 protein-coding genes across the 17 cancer types. Ultimately, these experiments identified prognostic genes for 17 different human cancer types. Prognostic genes were defined as genes for which the expression level above or below the experimentally determined cutoff in an individual patient yields a significant ($P < 0.001$) difference in patient's survival.

Genome-wide gene expression profiling experiments revealed that human cancer survival genes are markedly enriched among genes expression of which is significantly altered in human cancer cells treated with P-bi-TAT. P-bi-TAT therapy significantly alters the expression of thousands cancer survival genes.

EFFECT OF P-bi-TAT TREATMENT ON EXPRESSION OF HUMAN CANCER SURVIVAL GENES

There are two categories of human cancer survival predictor genes:

1) **Genes for which increased expression is significantly associated with favorable prognosis (increased likelihood of survival and decreased probability of death from cancer: longer patients survival defined as good outcome);**

2) **Genes for which increased expression is significantly associated with unfavorable prognosis (decreased likelihood of survival and increased probability of death from cancer: shorter patients survival defined as poor outcome);**

Our experiments identified gene expression signatures reflecting a transcriptional reversal of death from cancer phenotypes induced by the P-bi-TAT therapy: increased expression of genes associated with favorable clinical outcomes and decreased expression of genes associated with unfavorable clinical outcomes.

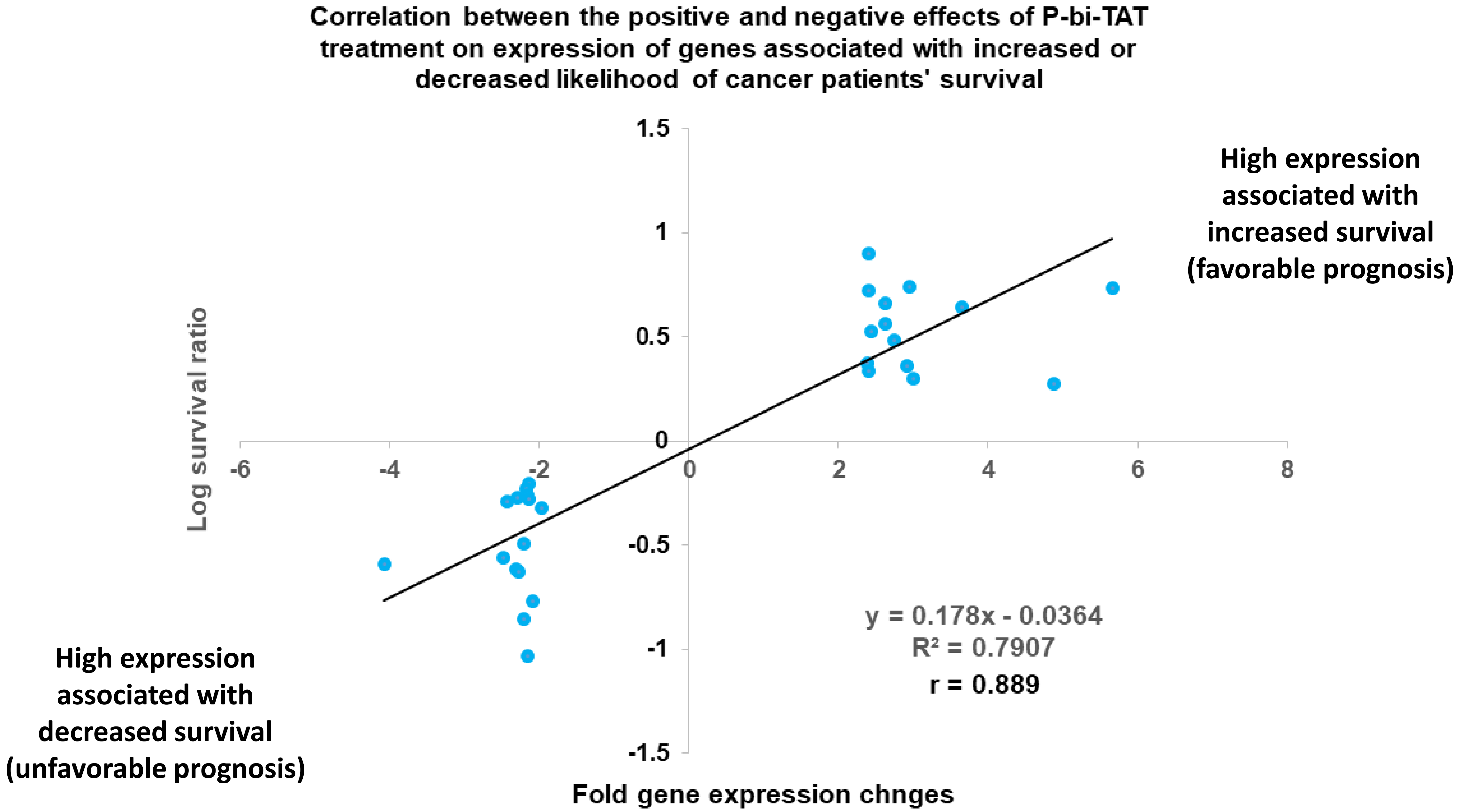

Correlation between the positive and negative effects of P-bi-TAT treatment on expression of genes associated with increased or decreased likelihood of cancer patients' survival
High expression associated with increased survival (favorable prognosis)
High expression associated with decreased survival (unfavorable prognosis)
Log survival ratio
1.5
1
0.5
0
-0.5
-1
-1.5
-6
-4
-2
0
2
4
6
8
y = 0.178x - 0.0364
R² = 0.7907
r = 0.889
Fold gene expression chnges

Effect of P-bi-TAT therapy on cancer survival predictor genes: increased expression of genes associated with favorable prognosis and decreased expression of genes associated with unfavorable clinical outcome

Gene Symbol	Fold Expression Change	P-value	Log Survival Ratio	Prognosis (cancer types)
HIBCH	2.39	0.018	0.902483	favorable (renal; ovarian)
AKR1B10	2.95	0.0135	0.74366	favorable (colorectal)
HIST1H1C	5.66	0.0089	0.736966	favorable (urothelial)
ARMT1	2.39	0.0242	0.723482	favorable (colorectal)
PIR	2.61	0.025	0.664899	favorable (cervical; ovarian)
CRYGS	3.64	0.0048	0.643856	favorable (urothelial)
DDX5	2.62	0.0017	0.566347	favorable (colorectal)
TNFRSF14	2.43	0.0108	0.527423	favorable (urothelial; endometrial; breast)
CSTA	2.73	0.0119	0.485427	favorable (head & neck)
AKR1B15	2.38	0.0032	0.378512	favorable (head & neck)
GCNT3	2.9	0.0039	0.364997	favorable (endometrial)
SEC61B	2.39	0.009	0.338802	favorable (endometrial)
DTNA	3	0.0002	0.303781	favorable (renal)
TIPARP	4.87	0.0385	0.276518	favorable (breast)
OCRL	-2.14	0.0033	-0.20249	unfavorable (breast)
TXNDC15	-2.18	0.0171	-0.22239	unfavorable (renal)
TCF20	-2.17	0.0047	-0.25716	unfavorable (liver)
SLC6A8	-2.3	0.0318	-0.26685	unfavorable (renal; thyroid)
MAGOH	-2.14	0.0226	-0.27462	unfavorable (renal)
SELL	-2.44	0.0279	-0.28728	unfavorable (renal)
EHD3	-1.97	0.0467	-0.31748	unfavorable (renal)
UTP14A	-2.22	0.0275	-0.48625	unfavorable (head & neck; renal)
APIP	-2.49	0.0092	-0.558	unfavorable (liver)
CD9	-4.08	0.0085	-0.58496	unfavorable (pancrreatic)
IGFBP3	-2.32	0.0253	-0.61444	unfavorable (colorectal; liver; renal)
RCC2	-2.28	0.0288	-0.62105	unfavorable (liver; renal; melanoma)
CEP250	-2.1	0.0263	-0.76332	unfavorable (liver; renal)
DHX35	-2.22	0.0008	-0.84812	unfavorable (liver; endometrial)
FUBP1	-2.16	0.0274	-1.02748	unfavorable (liver)

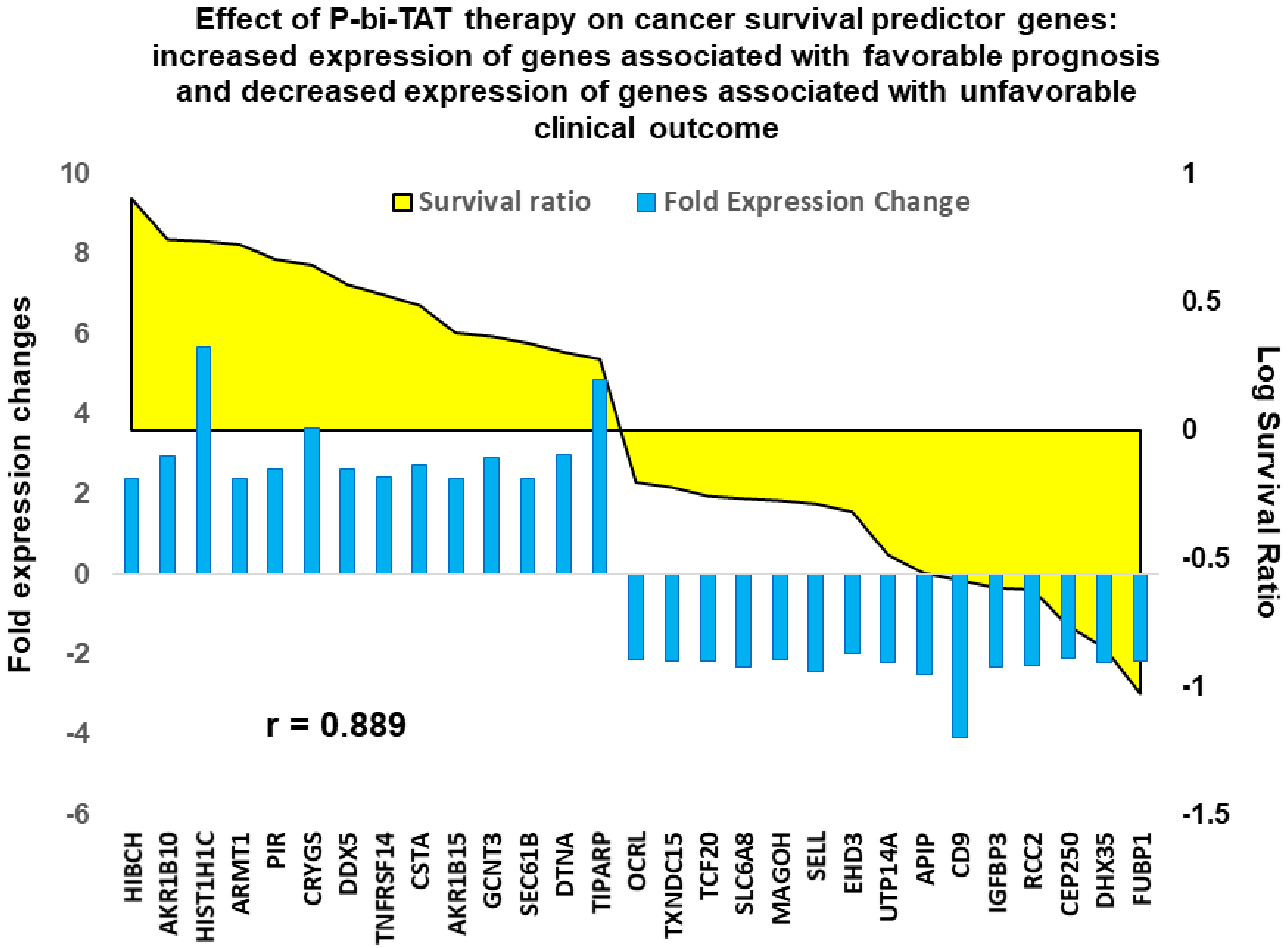
Effect of P-bi-TAT therapy on cancer survival predictor genes: increased expression of genes associated with favorable prognosis and decreased expression of genes associated with unfavorable clinical outcome
Survival ratio
Fold Expression Change
Fold expression changes
Log Survival Ratio
r = 0.889
10
8
6
4
2
0
-2
-4
-6
1
0.5
0
-0.5
-1
-1.5
HIBCH
AKR1B10
HIST1H1C
ARMT1
PIR
CRYGS
DDX5
TNFRSF14
CSTA
AKR1B15
GCNT3
SEC61B
DTNA
TIPARP
OCRL
TXNDC15
TCF20
SLC6A8
MAGOH
SELL
EHD3
UTP14A
APIP
CD9
IGFBP3
RCC2
CEP250
DHX35
FUBP1

Survival and expression agreements

Melanoma

UNG

MELANOMA - Survival analysis

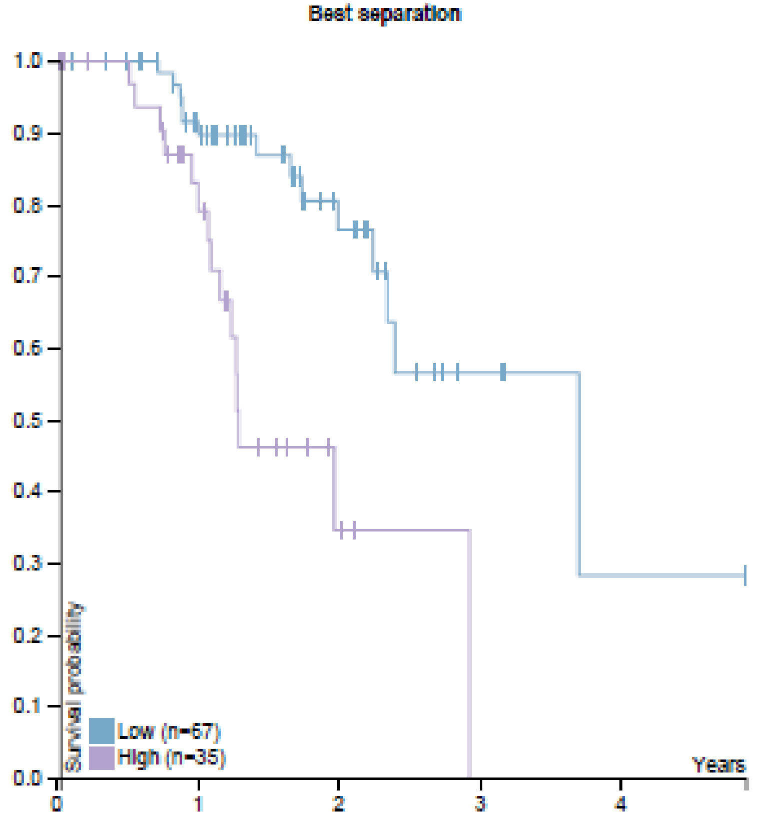

Expression cutoff	20.0 FPKM
3-year survival high	0%
3-year survival low	56%
Log-rank P value	3.06e-4 (Prognostic, unfavourable)

Down-regulated consensus

Survival and expression agreements

Glioma

SDC1

GLIOMA - Survival analysis

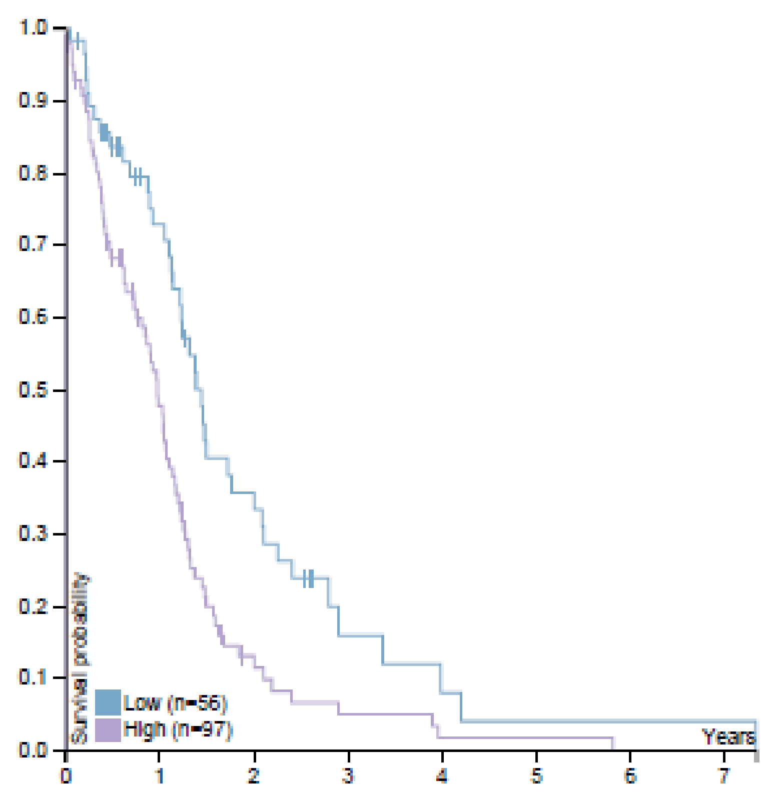

Expression cutoff	3.3 FPKM
3-year survival high	3%
3-year survival low	12%
Log-rank P value	8.05e-4 (Prognostic, unfavourable)

Down-regulated consensus

ZBTB6

GLIOMA - Survival analysis[i]

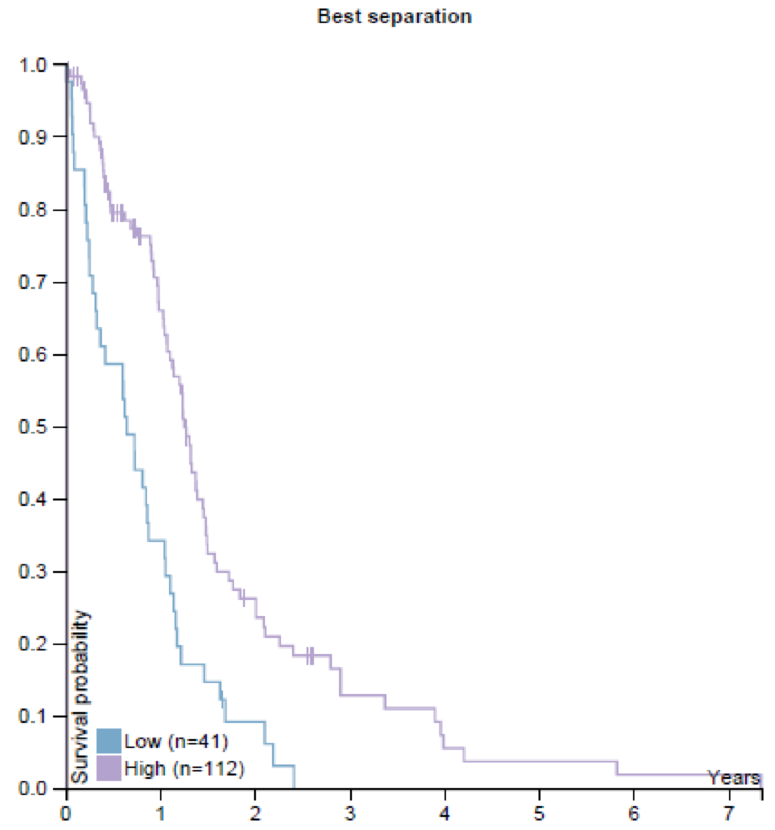

**Expression cutoff**[i]	3.6 FPKM
**3-year survival high**[i]	11%
**3-year survival low**[i]	0%
**Log-rank P value**[i]	9.75e-6 (Prognostic, favourable)

Up-regulated GBM

Examples from consensus signature

CONSENSUS SET OF 737 GENES AFFECTED BY P-bi-TAT THERAPY

Primary human glioblastoma cells **GBM 021913**

Human metastatic pancreatic cancer cell line **SUIT2-luc**

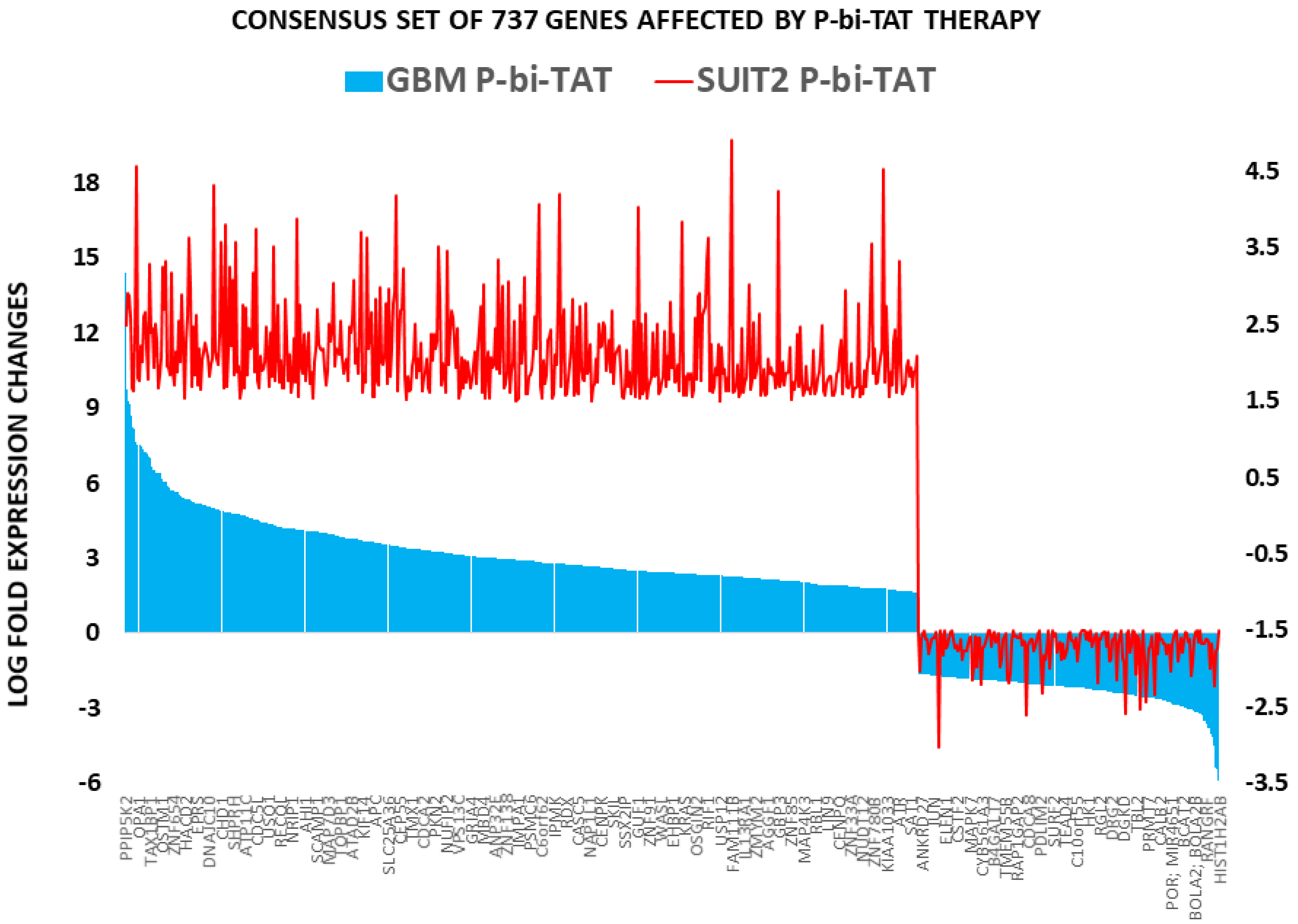
CONSENSUS SET OF 737 GENES AFFECTED BY P-bi-TAT THERAPY
GBM P-bi-TAT
SUIT2 P-bi-TAT
LOG FOLD EXPRESSION CHANGES
18
15
12
9
6
3
0
-3
-6
4.5
3.5
2.5
1.5
0.5
-0.5
-1.5
-2.5
-3.5

ZNF185

PANCREATIC CANCER - Survival analysis

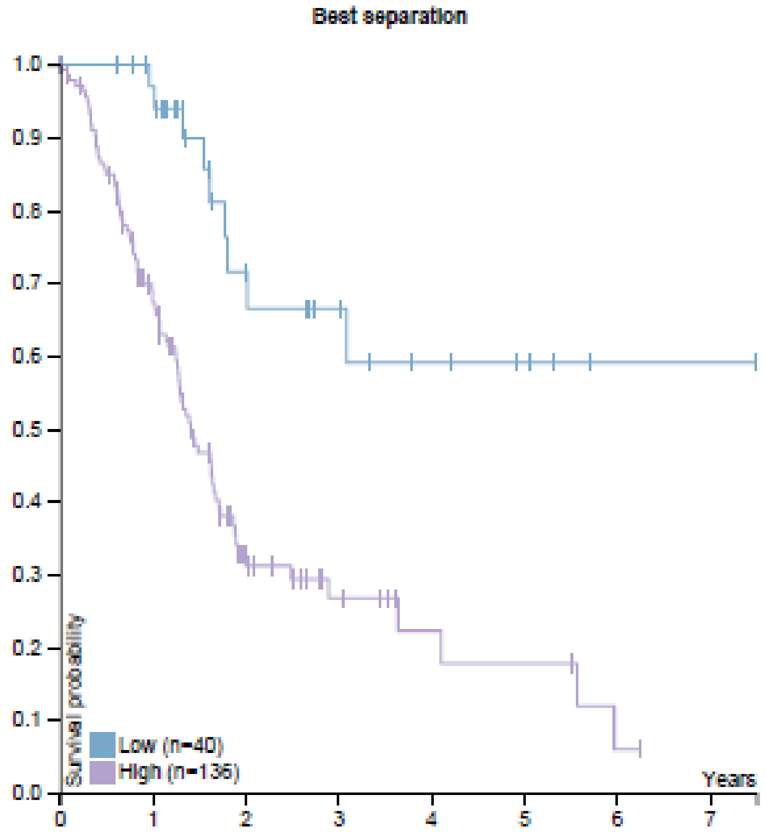

Expression cutoff	4.7 FPKM
5-year survival high	18%
5-year survival low	59%
Log-rank P value	3.30e-5 (Prognostic, unfavourable)

Down-regulated

UNG

MELANOMA - Survival analysis

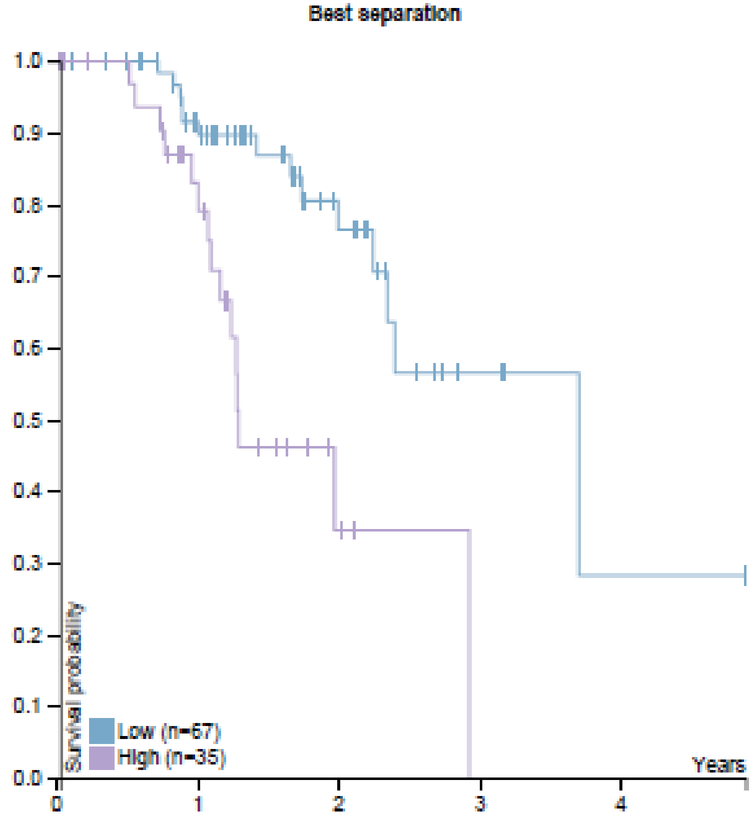

Expression cutoff	20.0 FPKM
3-year survival high	0%
3-year survival low	56%
Log-rank P value	3.06e-4 (Prognostic, unfavourable)

Down-regulated

SDC1

GLIOMA - Survival analysis

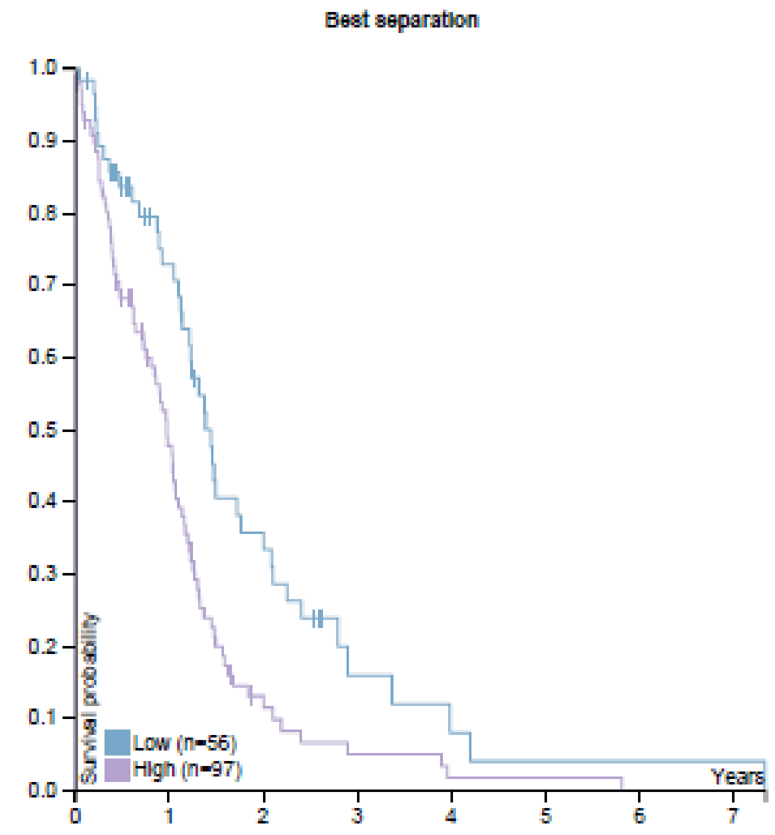

Expression cutoff	3.3 FPKM
3-year survival high	3%
3-year survival low	12%
Log-rank P value	8.05e-4 (Prognostic, unfavourable)

Down-regulated

HIST1H1C

PANCREATIC CANCER - Survival analysis

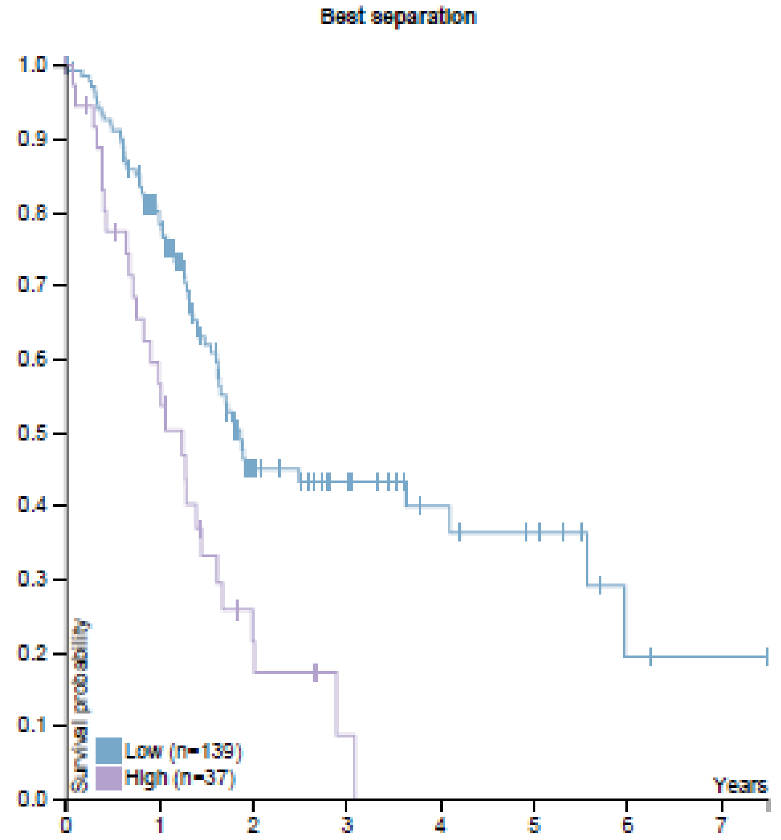

Expression cutoff	108.2 FPKM
5-year survival high	0%
5-year survival low	36%
Log-rank P value	1.12e-4 (Prognostic, unfavourable)

Down-regulated (GBM)

HIST1H3H

PANCREATIC CANCER - Survival analysis

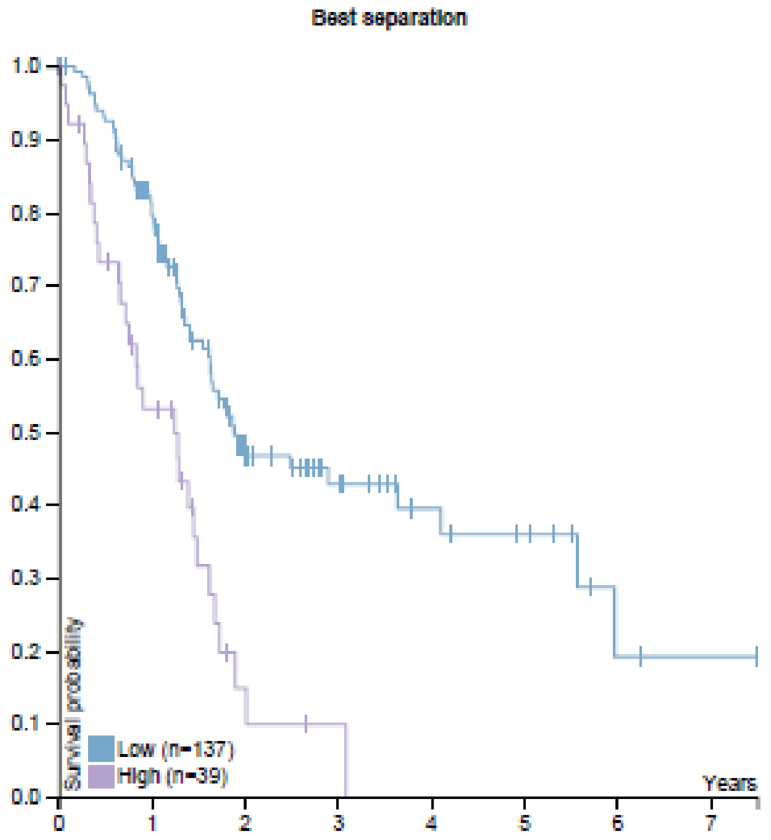

Expression cutoff	3.8 FPKM
5-year survival high	0%
5-year survival low	36%
Log-rank P value	9.25e-6 (Prognostic, unfavourable)

Down-regulated (GBM)
